# Supplementary material for: Hyperbolic metamaterial empowered controllable photonic Weyl nodal line semimetals
Source: Nat Commun. 2024 Mar 30;15:2773. doi: 10.1038/s41467-024-47125-7 (PMC10981722; doi:10.1038/s41467-024-47125-7)
Supplement: Supplementary file 1 — Supplementary Information [file 41467_2024_47125_MOESM1_ESM.pdf]

Supplementary Materials for "Hyperbolic Metamaterial Empowered Controllable Photonic Weyl Nodal Line Semimetals"

Shengyu Hu[1], Zhiwei Guo[1*], Wenwei Liu[2,3,4], Shuqi Chen[2,3,4,5], and Hong Chen[1]

1. *MOE Key Laboratory of Advanced Micro-Structured Materials, School of Physics Science and Engineering, Tongji University, Shanghai 200092, China*
2. *The Key Laboratory of Weak Light Nonlinear Photonics, Ministry of Education, School of Physics and TEDA Institute of Applied Physics, Nankai University, Tianjin 300071, China*
3. *Renewable Energy Conversion and Storage Center, Nankai University, Tianjin 300071, China*
4. *Smart Sensing Interdisciplinary Science Center, Nankai University, Tianjin 300071, China*
5. *The Collaborative Innovation Center of Extreme Optics, Shanxi University, Taiyuan 030006 Shanxi, China*

*Corresponding authors' Emails: 2014guozhiwei@tongji.edu.cn

Sec. I. EMT for HMM containing 2D material 2

Sec. II. DOF of q_2 4

Sec. III. DOF of ϕ and the critical condition 8

Sec. IV. The existence condition of bilateral drumhead surface state (DSS) 11

Sec. V. Singularities pairs and degenerate bound states in the continuum 17

Sec. VI. Berry curvature vortex near the nodal lines 23

Sec. VII. Strategy for dual-mode sensing 24

Sec. VIII. Absorption losses and annihilation of singularities 28

Sec. IX. Description of electromagnetic parameters 32

Sec. I. EMT for HMM containing 2D material

To unveil the feasibility and validity of EMT for HMM containing 2D material, we consider a metamaterial A with an (N+1)-layered subwavelength substructure, which is denoted by $\mathrm{A}^{(1)}\mathrm{A}^{(2)}\dots\mathrm{A}^{(L)}\mathrm{G}\mathrm{A}^{(L+1)}\dots\mathrm{A}^{(N-1)}\mathrm{A}^{(N)}$. Here a single layer G of the conductive sheet, such as graphene, is embedded between the Lth and $(L+1)^{\text{th}}$ bulk layers. Assuming each bulk layer is isotropic and nonmagnetic, i.e. $\mu = 1$, the constitutive equation for the jth bulk layer can be described as:

$$\begin{cases} D_x^{(j)} = \varepsilon_0 \varepsilon_j E_x^{(j)} \\ D_y^{(j)} = \varepsilon_0 \varepsilon_j E_y^{(j)}, \\ D_z^{(j)} = \varepsilon_0 \varepsilon_j E_z^{(j)} \end{cases} \tag{S1}$$

where ε_0 is the vacuum permittivity and ε_j is the relative permittivity of the jth bulk layer, respectively. In the chosen coordinate system, the z-axis is perpendicular to the propagation direction of the electromagnetic waves, hence components $E_x^{(j)}$, $E_y^{(j)}$ and $D_z^{(j)}$ are continuous according to Faraday's law $\nabla \times \boldsymbol{E} = -\frac{\partial}{\partial t}\boldsymbol{B}$. Taken into account the thicknesses of the bulk layers are much smaller than the incident wavelength ($d_j \ll \lambda$), the above-mentioned electrical parameters inside the bulk layers can be regarded as uniform, namely

$$\begin{cases} \langle E_x \rangle = E_x^{(1)} = E_x^{(2)} = \cdots = E_x^{(N)} \\ \langle E_y \rangle = E_y^{(1)} = E_y^{(2)} = \cdots = E_y^{(N)}, \\ \langle D_Z \rangle = D_Z^{(1)} = D_Z^{(2)} = \cdots = D_Z^{(N)} \end{cases} \tag{S2}$$

where $\langle E_{x,y} \rangle = \frac{1}{d}\sum_{j=1}^{N} E_{x,y}^{(j)} d_j$ as well as $\langle D_z \rangle = \frac{1}{d}\sum_{j=1}^{N} D_Z d_j$ denotes the mean parameters over the whole metamaterial, and $d = \sum_{j=1}^{N} d_j$ denotes the thickness of the unit cell. In that case, the other parameters can be expressed in terms of the continuous ones [S1]:

$$\begin{cases} D_x^{(j)} = \varepsilon_0 \varepsilon_j E_x^{(j)} \\ D_y^{(j)} = \varepsilon_0 \varepsilon_j E_y^{(j)} \\ E_z^{(j)} = \frac{1}{\varepsilon_0 \varepsilon_j} D_z^{(j)} \end{cases}. \tag{S3}$$

On the other hand, when electromagnetic waves propagate through the interface formed on the conductive sheet G, the surface current density of the conductive sheet can be described as $J_{x,y} = \sigma_G E_{x,y}$ based on Ohm's law [S2]. Here σ_G is the conductivity of the sheet. Combining with Ampere-Maxwell law $\nabla \times \boldsymbol{H} = \frac{\partial}{\partial t}\boldsymbol{D} + \boldsymbol{J}$, the contribution of J_x can be attributed to the effective electric displacement $D_{x,y}^{eff} = \frac{i\sigma E_{x,y}}{\omega}$ with time dependence $e^{-i\omega t}$ [S3]. Combining with Eqs. (S2) and (S3), we ultimately get the effective constitutive equation for the whole metamaterial:

$$\begin{cases} \langle D_x \rangle d = \sum_{j=1}^{N} \varepsilon_0 \varepsilon_j E_x^{(j)} d_j + D_x^{eff} = \langle E_x \rangle (\sum_{j=1}^{N} \varepsilon_0 \varepsilon_j d_j + \frac{i\sigma}{\omega}) \\ \langle D_y \rangle d = \sum_{j=1}^{N} \varepsilon_0 \varepsilon_j E_y^{(j)} d_j + D_y^{eff} = \langle E_y \rangle (\sum_{j=1}^{N} \varepsilon_0 \varepsilon_j d_j + \frac{i\sigma}{\omega}) \\ \langle E_Z \rangle d = \sum_{j=1}^{N} \frac{1}{\varepsilon_0 \varepsilon_j} D_Z^{(j)} d_j = \langle D_Z \rangle (\sum_{j=1}^{N} \frac{d_j}{\varepsilon_0 \varepsilon_j}) \end{cases}, \tag{S4}$$

and the components of the effective permittivity tensor are given by:

$$\begin{cases} \varepsilon_{\parallel} = \varepsilon_{\mathrm{A}x} = \varepsilon_{\mathrm{A}y} = \sum_{j=1}^{N} \varepsilon_j \delta_j + \frac{i\sigma}{\varepsilon_0 \omega d} \\ \varepsilon_{\perp} = \varepsilon_{\mathrm{A}z} = 1/(\sum_{j=1}^{N} \delta_j / \varepsilon_j) \end{cases}, \tag{S5}$$

where $\delta_j = d_j/d$ is the filling ratio of the jth bulk layer. Obviously, introducing 2D material into HMM will only have an effect on the radial effective permittivity.

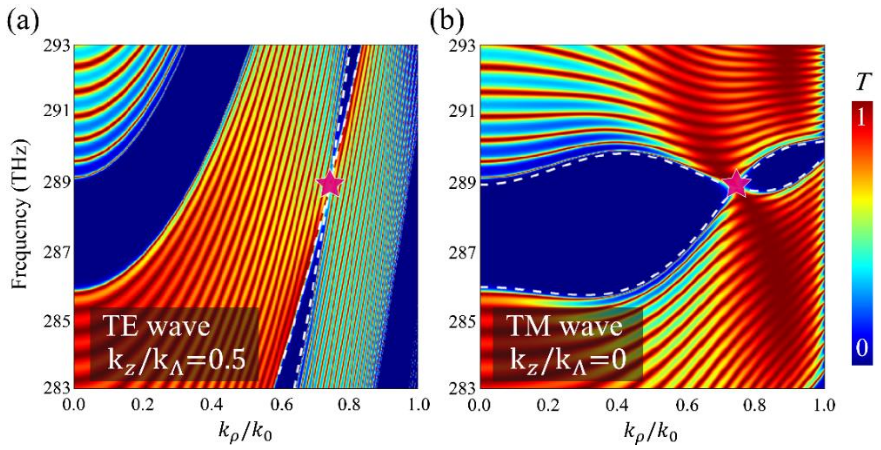

FIG. S1. Based on EMT, the transmission spectra of 1D hyper-crystal $(AB)_{20}$ for (a) TE and (b) TM waves. The parameters are identical to those in Fig. 2(a).

Subsequently, the effectiveness of the above method is demonstrated. The transmission spectra of 1D hyper-crystal $[AB]_{20}$ for TE and TM modes are shown in Figs. S1(a) and S1(b), respectively. Here the thicknesses of layers A and B are $d_{\mathrm{A}} = 1210$ nm and $d_{\mathrm{B}} = 2696$ nm. Other parameters are the same as those in Fig. 2. Note that the effective parameter is relevant to the thickness of the unit cell for HMM A according to Eq. (S5). It can be seen that degenerate points for two polarizations appear at $f_0 = 288.8$ THz and $k_{\rho 0}/k_0 = 0.74$ simultaneously, which are shown by the stars. Meanwhile, the former exists at the zone boundary ($k_z/k_\Lambda = 0.5$), while the latter exists at the zone center ($k_z/k_\Lambda = 0$). Thus, this configuration corresponds to the quasi-DNLS mentioned in the main text. As a comparison, figures. 2(c) and 2(d) provide the spectra of 1D hyper-crystal $[(CDE)_{22}B]_{20}$, which can meet with the above results of EMT basically. This conclusion provides a concise and convenient way to design the nodal line semimetal in the system of optics.

Sec. II. DOF of q_2

In the main text, the Fermi energy of the graphene shifts the crossing point along the axis of f and k_ρ, which provides the DOFs of Δ and q_1 in Eq. (2). Meanwhile, our previous study demonstrates that the thicknesses of component layers can modulate the DOF of q_2 and even ϕ [S4]. In this section, we will theoretically prove this relationship. Without consideration of the boundary effect, the band structure of the discussed 1D PC with infinite periods ($N \to \infty$) can be given by

$$\cos(k_z\Lambda) = \cos(k_{\mathrm{Az}}d_{\mathrm{A}})\cos(k_{\mathrm{Bz}}d_{\mathrm{B}}) - \frac{1}{2}\left(\frac{\eta_{\mathrm{Az}}}{\eta_{\mathrm{Bz}}} + \frac{\eta_{\mathrm{Bz}}}{\eta_{\mathrm{Az}}}\right)\sin(k_{\mathrm{Az}}d_{\mathrm{A}})\sin(k_{\mathrm{Bz}}d_{\mathrm{B}}) \quad ,$$

(S6)

where k_z denotes the Bloch wave vector, and $\Lambda = d_{\mathrm{A}} + d_{\mathrm{B}}$ denotes the length of the unit cell. The impedance η_{iz} $(i = \mathrm{A}, \mathrm{B})$ will be affected by the polarizations, that is $\eta_{iz}^{\mathrm{TE}} = \frac{\mu_{ix}}{n_{iz}^{\mathrm{TE}}}$ for the TE wave as well as $\eta_{iz}^{\mathrm{TM}} = \frac{n_{iz}^{\mathrm{TM}}}{\varepsilon_{ix}}$ for the TM wave. Especially, $\tilde{n}_i^{\mathrm{TE}} = k_{iz}^{\mathrm{TE}}/k_0 = \sqrt{\varepsilon_{iy}\mu_{ix} - \frac{\mu_{ix}k_\rho^2}{\mu_{iz}k_0^2}}$ and $\tilde{n}_i^{\mathrm{TM}} = k_{iz}^{\mathrm{TM}}/k_0 = \sqrt{\varepsilon_{ix}\mu_{iy} - \frac{\varepsilon_{ix}k_\rho^2}{\varepsilon_{iz}k_0^2}}$ $(i = \mathrm{A}, \mathrm{B})$ are the effective refractive indexes for TE and TM modes, respectively. At a special point $(f_0^\iota, k_{\rho 0}^\iota)$, the accidental degeneracies tend to occur with the condition for the ratio of optical paths

$$\alpha^\iota = \frac{\tilde{n}_{\mathrm{A}}^\iota d_{\mathrm{A}}}{\tilde{n}_{\mathrm{B}}^\iota d_{\mathrm{B}}} = \frac{m^\iota}{n^\iota} \epsilon \mathbb{Q}, \quad \text{(S7)}$$

where $\{m^\iota, n^\iota\} \epsilon \mathbb{Z}^+$, and the superscript ι represents the TE or TM polarization [S5]. This crossing band structure can be perceived as a single WNLS. And we can further get the frequency $f_0^\iota = \frac{(m^\iota + n^\iota)c}{2(\tilde{n}_{\mathrm{A}}^\iota d_{\mathrm{A}} + \tilde{n}_{\mathrm{B}}^\iota d_{\mathrm{B}})}$ of its Fermi energy Δ. Linked with such a fixed point $(f_0^\iota, k_{\rho 0}^\iota)$, this parameter group of thicknesses can be denoted as $(d_{\mathrm{A}}, d_{\mathrm{B}})$, and each element in the set $\{(P^\iota d_{\mathrm{A}}/m^\iota, Q^\iota d_{\mathrm{B}}/n^\iota) | \{P^\iota, Q^\iota\} \epsilon \mathbb{Z}^+\}$ will also meet the rational condition in Eq. (S7) simultaneously, which turns into $\alpha_{\mathrm{new}}^\iota = \frac{P^\iota}{Q^\iota} \epsilon \mathbb{Q}$ naturally. In that case, the modulation of the thicknesses can be exactly seen as a DOF [S4].

At present, let us come back to the origin $(d_{\mathrm{A}}, d_{\mathrm{B}})$ of the evolution. At the degenerate point $(f_0^\iota, k_{\rho 0}^\iota)$, the propagation phase can be described as $k_{\mathrm{A}z}^\iota d_{\mathrm{A}} = m^\iota \pi$ and $k_{\mathrm{B}z}^\iota d_{\mathrm{B}} = n^\iota \pi$ in the component layers A and B, respectively. And the amplitudes of the incident and outgoing waves are the same or completely opposite when $(m^\iota + n^\iota) mod2 = 0$ or $(m^\iota + n^\iota) mod2 = 1$, and the degenerate point occurs at the zone center $(k_z/k_\Lambda = 0)$ or zone boundary $(k_z/k_\Lambda = 0.5)$, respectively. Therefore, the

optical properties near the degenerate point are similar to those of epsilon-near-zero (ENZ) or mu-near-zero (MNZ) materials to some extent. Meanwhile, some designed structures, 1D PCs with isotropic component layers in particular, are able to meet the condition for both TE and TM waves at the same point $(f_0, k_{\rho 0})$, which lays a theoretical foundation for the phases of degenerate Weyl nodal lines, including distinguished DNLSs and so-called quasi-DNLSs. Sometimes, there exists such a case: $(m^{\mathrm{TE}} + n^{\mathrm{TE}}) mod2 \neq (m^{\mathrm{TM}} + n^{\mathrm{TM}}) mod2$, just like the structure in Fig. 2(a). For photonic quasi-DNLS with actual structure $[(CGD)_{22}B]_{20}$ illustrated in Figs. 2(c) and 2(d), the concerned regions near the degenerate points are highlighted in Figs. S2(a) and S2(c). In the normalized parameter space of $E - k_\rho - k_z$, figures S2(b) and S2(d) indicate the band structures have linear dispersions along directions of k_ρ and k_z, which are respectively marked by green and red lines. In that case, two WNLSs for different polarizations are separate along the axis of k_z, which corresponds to the phase of quasi-DNLSs.

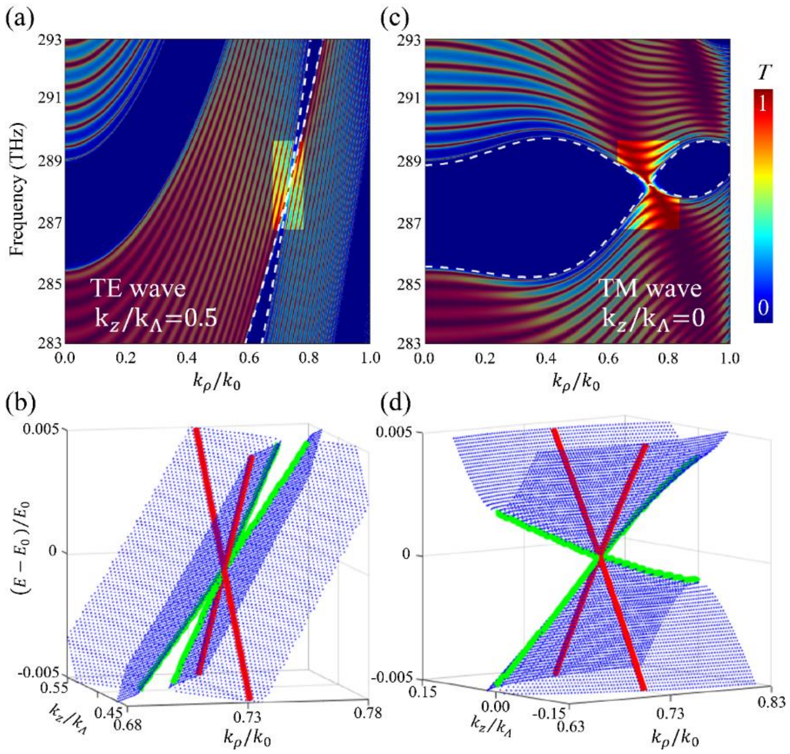

FIG. S2. A photonic quasi-DNLS. (a) Transmission spectra of the actual structure $[(CGD)_{22}B]_{20}$ for the TE wave with band edges $k_z/k_\Lambda = 0.5$ (white dotted lines). The concerned region near the degenerate point is highlighted, while the other part is partially opaque. (b) Corresponding band structure of the concerned region in the normalized parameter space of $E - k_\rho - k_z$. The linear dispersions are marked by green and red lines along the directions of k_ρ and k_z, respectively. (c), (d) Similar to (a), (b), but for the TM wave. Noteworthy, band edges turn into $k_z/k_\Lambda = 0$ at this time, and this difference between polarizations leads to quasi-DNLSs.

Assuming each thickness d_A or d_B is doubled, $P^\iota = 2m^\iota$ as well as $Q^\iota = 2n^\iota$ decides $(P^\mathrm{TE} + Q^\mathrm{TE})mod2 = (P^\mathrm{TM} + Q^\mathrm{TM})mod2 = 0$, like $m^\mathrm{TE} = 7$, $m^\mathrm{TM} = 8$, $n^\mathrm{TE} = n^\mathrm{TM} = 8$, and $P^\mathrm{TE} = 14$, $P^\mathrm{TM} = 16$, $Q^\mathrm{TE} = Q^\mathrm{TM} = 16$ in Fig. S3. That is $d_{\mathrm{A}'} = 2d_\mathrm{A}$ and $d_{\mathrm{B}'} = 2d_\mathrm{B}$. Noteworthy, taking the effectiveness of Eq. (3) into account, $d_{A'}$ here is regulated by changing the period number S of the HMM layer A, that is *S'=2S*=44. In this situation, the parities of $P^\mathrm{TE} + Q^\mathrm{TE}$ and $P^\mathrm{TM} + Q^\mathrm{TM}$ become identical, which imply the similar band edges of $k_z/k_\Lambda = 0$ with white dotted lines in Figs. S3(a) and S3(b). And this band structure corresponds to the phase of DNLSs. Interestingly, there exist extra degenerate points W_2 and W_3 near the working

Fermi surface for the TM wave. $W_2 = \left(f_0 = 287.63\ \text{THz}, k_{\rho 0}/k_0 = 0.4689\right)$ corresponds to $P^{\text{TM}} = 15$ and $Q^{\text{TM}} = 17$, while $W_3 = (f_0 = 288.8\ \text{THz}, k_{\rho 0}/k_0 = 0.9144)$ corresponds to $P^{\text{TM}} = 17$ and $Q^{\text{TM}} = 15$. That is two WNLSs gather at the zone center ($k_z/k_\Lambda = 0$), which corresponds to the phase of DNLSs. In that case, we double the thicknesses of the component layers A and B at the same time to realize the phase transition from quasi-DNLSs to DNLSs. Namely, the modulation of thicknesses can directly impact on the DOF of q_2 in Eq. (2).

Sec. III. DOF of ϕ and the critical condition

In this section, we will further reveal the potential relationship between the thicknesses and the DOF of ϕ, which can control phase transitions among Type-I/II/III in the system of double WNLSs. To avoid the redundant discussion about the dimension of k_z, the following exploration is based on the frame of DNLSs in this section, like the structure in Fig. S3. Without loss of generality, similar conclusions can also be obtained in the systems of quasi-DNLSs and even other isolated WNLSs. In some circumstances, the component layers A and B have different physical properties, such as group velocity. For example, the HMM for the layer A provides a negative group velocity, while the dielectric for the layer B provides a positive group velocity. With the change of the ratio of the thicknesses, the group velocity of the whole 1D PC is able to be modulated almost continuously within a scope, which induces the novel phase transitions among Type-I/II/III WNLSs. Considering the HMM A is electrical in the main text, such phase transitions occur primarily for the TM mode. $(P^{\text{TM}} = 16, Q^{\text{TM}} = 16)$ corresponds to $d_{\text{A}'} = \frac{P^{\text{TM}}}{m^{\text{TM}}} d_{\text{A}} = 2d_{\text{A}}$ together with $d_{\text{B}'} = \frac{Q^{\text{TM}}}{n^{\text{TM}}} d_{\text{B}} = 2d_{\text{B}}$, as

shown in Fig. 2(j). Noteworthy, taking the effectiveness of Eq. (S5) into account, $d_{\mathrm{A}'}$ here is regulated by changing the period number S of the HMM layer A, that is S'=2S=44. By chance, this thickness ratio meets the phase variation compensation effect for the TM mode at the frequency $f_0 = 288.8$ THz:

$$\frac{d_{\mathrm{B}'}}{d_{\mathrm{A}'}} = \frac{d_{\mathrm{B}}}{d_{\mathrm{A}}} = -\frac{\sqrt{\varepsilon_{\parallel}\varepsilon_{\mathrm{B}}/\mu_{\parallel}}}{\varepsilon_{\perp}}. \quad \text{(S8)}$$

In other words, the Bragg condition is satisfied at this frequency for arbitrary k_ρ:

$$\left(k_{\mathrm{A}z}^{\mathrm{TM}} d_{\mathrm{A}'} + k_{\mathrm{B}z}^{\mathrm{TM}} d_{\mathrm{B}'}\right)\Big|_{f_0} = \left(P^{\mathrm{TM}} + Q^{\mathrm{TM}}\right)\pi, \quad \text{(S9)}$$

and the middle frequency of the band gap matches the Fermi energy Δ of the WNLS.

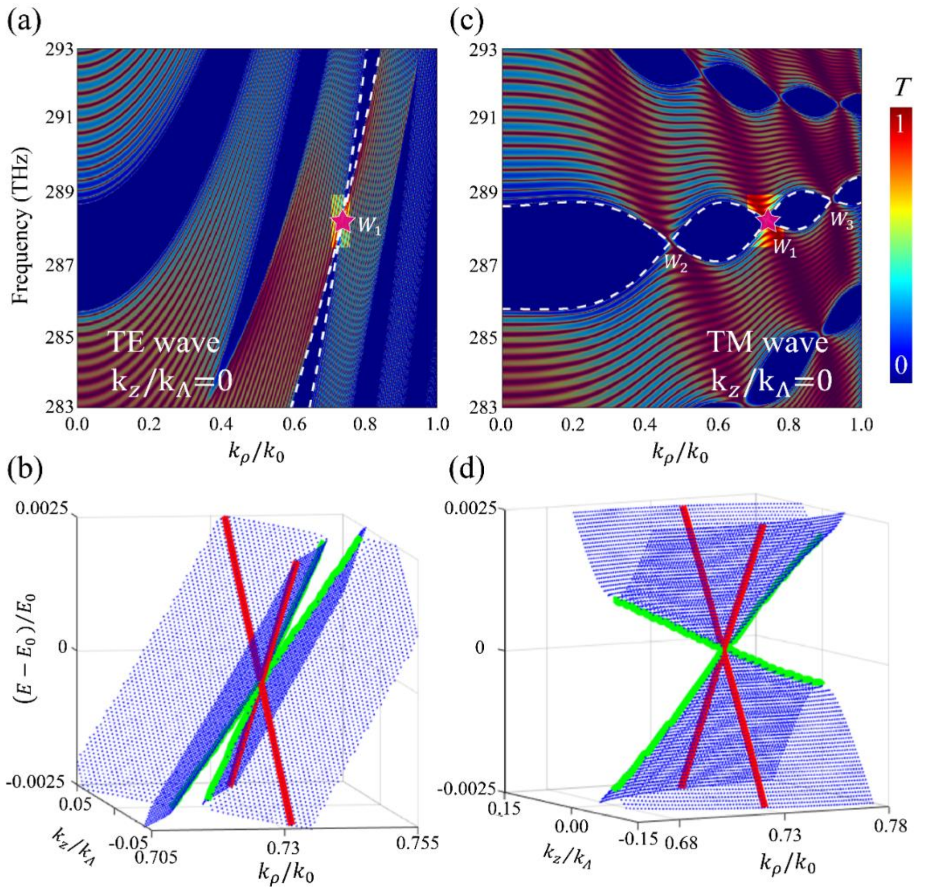

FIG. S3. A photonic DNLS. (a) The transmission spectrum of the actual structure [(CGD)$_{44}$B']$_{20}$ for the TE wave with band edges $k_z/k_\Lambda = 0$ (white dotted lines). Compared with the structure in Fig. 2 (a), $d_{\mathrm{B}'} = 2d_{\mathrm{B}}$ here, and other parameters remain unchanged. Similarly, the concerned region near the degenerate point W$_1$ (white star) is highlighted, while the other part is partially opaque. (b) Corresponding band structure of the concerned region in the normalized parameter space of $E - k_\rho - k_z$. The linear dispersions are marked by green and red lines along the directions

of k_ρ and k_z, respectively. (c), (d) Similar to (a), (b), but for the TM wave. At this time, the band structure near W1 can be perceived as a photonic DNLS. And two extra degenerate points W_2 and W_3 exist in the same band for the TM wave in (c).

Subsequently, an interesting question is whether this fascinating critical angle ϕ can be described by a certain ratio of thicknesses. Here we make an attempt to obtain an analytic interpretation. We consider an infinitesimal shift Δk along the axis of k_ρ near the degenerate point $(f_0, k_{\rho 0})$. According to the situation of phase variation compensation, we take $k_{\mathrm{Az}}^{\mathrm{TM}}(f_0, k_{\rho 0} + \Delta k) d_{\mathrm{A}} = m^{\mathrm{TM}}\pi + \Delta\varphi$ and $k_{\mathrm{Bz}}^{\mathrm{TM}}(f_0, k_{\rho 0} + \Delta k) d_{\mathrm{B}} = n^{TM}\pi - \Delta\varphi$, where $\Delta\varphi$ is the infinitesimal increment of the propagation phase owing to Δk, and they have the same sign. Then we adjust the thickness ratio $(d_{\mathrm{A}}, d_{\mathrm{B}}) \rightarrow \left(\frac{P^{\mathrm{TM}}}{m^{\mathrm{TM}}} d_{\mathrm{A}}, \frac{Q^{\mathrm{TM}}}{n^{\mathrm{TM}}} d_{\mathrm{B}}\right) = (d_{\mathrm{A}'}, d_{\mathrm{B}'})$ to modulate the tilt angle ϕ of the cone-like band. At present, $k_{\mathrm{Az}}^{\mathrm{TM}}(f_0, k_{\rho 0} + \Delta k) d_{\mathrm{A}'} = P^{\mathrm{TM}}\pi + \frac{P^{\mathrm{TM}}}{m^{\mathrm{TM}}}\Delta\varphi$ and $k_{\mathrm{Bz}}^{\mathrm{TM}}(f_0, k_{\rho 0} + \Delta k) d_{B'} = Q^{\mathrm{TM}}\pi - \frac{Q^{\mathrm{TM}}}{n^{\mathrm{TM}}}\Delta\varphi$. By combining with Eq. (S6), we can get the band structure with the highest order approximation:

$$\cos\left(k_z(f_0, k_{\rho 0} + \Delta k)\Lambda'\right) = \cos\left(P^{\mathrm{TM}}\pi + \frac{P^{\mathrm{TM}}}{m^{\mathrm{TM}}}\Delta\varphi\right)\cos\left(Q^{\mathrm{TM}}\pi - \frac{Q^{\mathrm{TM}}}{n^{\mathrm{TM}}}\Delta\varphi\right) - \frac{1}{2}\left(\frac{\eta_{\mathrm{Az}}}{\eta_{\mathrm{Bz}}} + \frac{\eta_{\mathrm{Bz}}}{\eta_{\mathrm{Az}}}\right)\sin\left(Q^{\mathrm{TM}}\pi - \frac{Q^{\mathrm{TM}}}{n^{\mathrm{TM}}}\Delta\varphi\right)\sin\left(Q^{\mathrm{TM}}\pi - \frac{Q^{\mathrm{TM}}}{n^{\mathrm{TM}}}\Delta\varphi\right) = (-1)^{P^{\mathrm{TM}}+Q^{\mathrm{TM}}}\left[1 - \frac{1}{2}\left(\frac{P^{\mathrm{TM}}}{m^{\mathrm{TM}}}\Delta\varphi\right)^2 - \frac{1}{2}\left(\frac{Q^{\mathrm{TM}}}{n^{\mathrm{TM}}}\Delta\varphi\right)^2 + \frac{1}{2}\left(\frac{\eta_{\mathrm{Az}}}{\eta_{\mathrm{Bz}}} + \frac{\eta_{\mathrm{Bz}}}{\eta_{\mathrm{Az}}}\right)\left(\frac{P^{\mathrm{TM}}}{m^{\mathrm{TM}}}\Delta\varphi\right)\left(\frac{Q^{\mathrm{TM}}}{n^{\mathrm{TM}}}\Delta\varphi\right)\right], \quad \text{(S10)}$$

where $\Lambda' = d_{\mathrm{A}'} + d_{\mathrm{B}'}$ is the recent unit cell length after adjustment. Assuming the group $(d_{A'}, d_{B'})$ conforms to the critical tilt angle of Type-III WNLSs correctly, the point $(f_0, k_{\rho 0} + \Delta k)$ should correspond to a band edge, that is $\cos\left(k_z(f_0, k_{\rho 0} + \Delta k)\Lambda'\right) = (-1)^{P^{\mathrm{TM}}+Q^{\mathrm{TM}}}$. Finally, we get the critical condition:

$$\left(\frac{P^{\mathrm{TM}}}{m^{\mathrm{TM}}}\right)^2 + \left(\frac{Q^{\mathrm{TM}}}{n^{\mathrm{TM}}}\right)^2 - \left(\frac{\eta_{\mathrm{Az}}}{\eta_{\mathrm{Bz}}} + \frac{\eta_{\mathrm{Bz}}}{\eta_{\mathrm{Az}}}\right)\left(\frac{P^{\mathrm{TM}}}{m^{\mathrm{TM}}}\right)\left(\frac{Q^{\mathrm{TM}}}{n^{\mathrm{TM}}}\right) = 0. \quad \text{(S11)}$$

Meanwhile, the tilt angles almost remain unchanged with the change of the

thickness ratio, as is shown in Fig. S4 due to the positive group velocity of both the layer A and B for the TE mode. In that case, we can realize a phase transition of the hybrid DNLS.

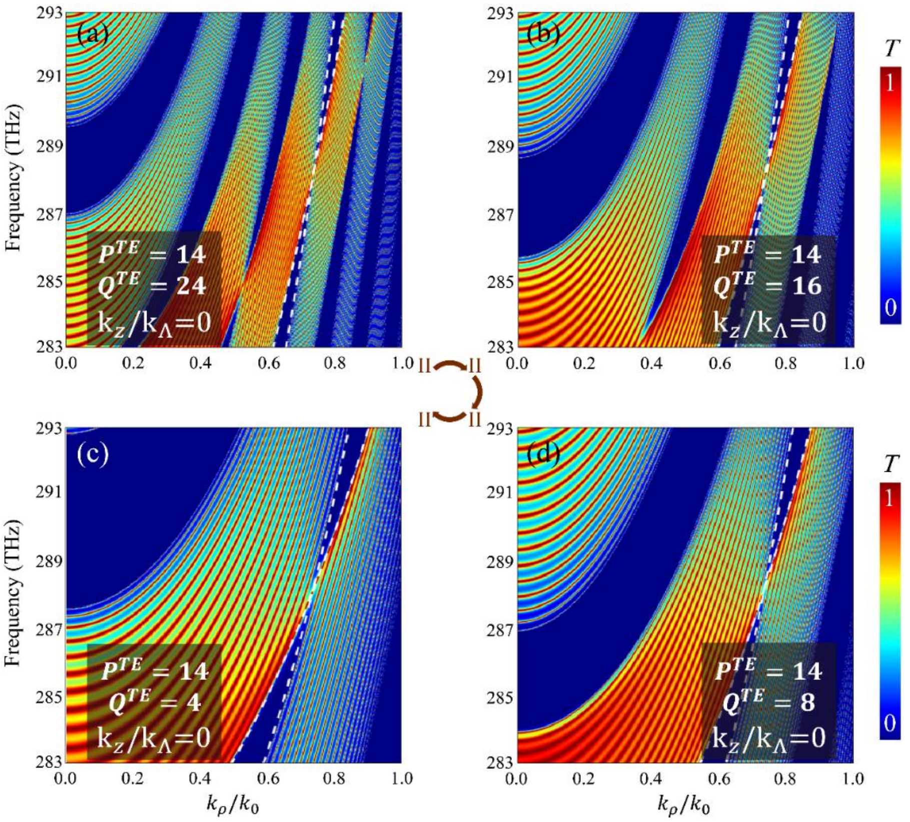

FIG. S4. For TE waves, the transmittance spectra of 1D hyper-crystal [(CGD)$_{44}$B']$_{20}$ with different thickness factors: (a) $P^{\mathrm{TE}} = 14, Q^{\mathrm{TE}} = 24$; (b) $P^{\mathrm{TE}} = 14, Q^{\mathrm{TE}} = 16$; (c) $P^{\mathrm{TE}} = 14, Q^{\mathrm{TE}} = 4$; (d) $P^{\mathrm{TE}} = 14, Q^{\mathrm{TE}} = 8$. Here $d_{\mathrm{B'}} = \frac{Q^{\mathrm{TE}}}{n^{\mathrm{TE}}} d_{\mathrm{B}}$, where $n^{\mathrm{TE}} = 8$ and $d_{\mathrm{B}} = 2696$ nm. The tilt angles of the conical bands almost remain unchanged, corresponding to type-II WNLSs.

Sec. IV. The existence condition of bilateral drumhead surface state (DSS)

For supergiant PCs, it is normally challenging to receive all of their physical parameters analytically due to the complex calculations with the huge number of unit cells. Typically, the boundary effect can be regarded as a perturbation to be neglected, and the energy band structure under periodic boundary condition (PBC) is quite similar to that under open boundary condition (OBC), which is the conventional bulk-edge correspondence principle. By the way, in light of practical applications, the boundary

potential barriers tend to be not so high. But the conclusion still retains. Through the band structure of one unit cell in PC like Eq. (S6), the properties of the total PC can be estimated, and the calculation process can also be greatly simplified. However, when the PC is truncated by another structure, it is generally difficult to utilize such a classical method, especially for the discussion of surface states existed in the interfaces. In this section, we will take the classical structure of metal-PC with Tamm-like surface states as an example to demonstrate a semi-analytic method based on the extracted reflection phases.

Considering the structure $[(\mathrm{CGD})_\mathrm{S}\mathrm{B}]_\mathrm{N}$ illustrated in Fig. 2(a), the correlation between the input and output magnetic fields can be described as:

$$\begin{pmatrix} H_{\mathrm{in}}^{+} \\ H_{\mathrm{in}}^{-} \end{pmatrix} = M_{\mathrm{PC}} \begin{pmatrix} H_{\mathrm{out}}^{+} \\ H_{\mathrm{out}}^{-} \end{pmatrix}, \tag{S12}$$

where the superscript + (-) denotes the forward (backward) component of the magnetic field strength, and $M_{\mathrm{PC}} = F_{\mathrm{in}}^{-1}[(F_{\mathrm{C}}P_{\mathrm{C}}U_{\mathrm{G}}P_{\mathrm{D}}F_{\mathrm{D}}^{-1})^{S}F_{\mathrm{B}}P_{\mathrm{B}}F_{\mathrm{B}}^{-1}]^{N}F_{\mathrm{out}}$ is the total transfer matrix of the 1D PC. The background is air, and $F_{\mathrm{in}} = F_{\mathrm{out}} = F_{0}$. Worth mentioning, $F_j = \begin{pmatrix} 1 & 1 \\ N_j & -N_j \end{pmatrix}$ is the interface matrix, $U_{\mathrm{G}} = \frac{1}{2}\begin{pmatrix} 1+\eta+\xi & 1-\eta-\varsigma\xi \\ 1-\eta+\varsigma\xi & 1+\eta-\xi \end{pmatrix}$ is the interface matrix with the influence of the graphene sheet G [S2], and $P_j = \begin{pmatrix} e^{-ik_{jz}d_j} & 0 \\ 0 & e^{ik_{jz}d_j} \end{pmatrix}$ is the propagating matrix $(j = 0, \mathrm{C}, \mathrm{D}, \mathrm{B})$. The polarization of the incident waves should be considered, that is $N_j^{\mathrm{TE}} = \frac{k_{jz}^{\mathrm{TE}}}{\omega\mu_0\mu_j}$ together with $N_j^{\mathrm{TM}} = \frac{k_{jz}^{\mathrm{TM}}}{\omega\varepsilon_0\varepsilon_j}$, $\eta^{\mathrm{TE}} = \frac{\mu_{\mathrm{C}}k_{\mathrm{D}z}^{\mathrm{TE}}}{\mu_{\mathrm{D}}k_{\mathrm{C}z}^{\mathrm{TE}}}$ together with $\eta^{\mathrm{TM}} = \frac{\varepsilon_{\mathrm{C}}k_{\mathrm{D}z}^{\mathrm{TM}}}{\varepsilon_{\mathrm{D}}k_{\mathrm{C}z}^{\mathrm{TM}}}$, $\xi^{\mathrm{TE}} = \frac{\sigma_{\mathrm{G}}\omega\mu_0\mu_{\mathrm{C}}}{k_{\mathrm{C}z}^{\mathrm{TE}}}$ together with $\xi^{\mathrm{TM}} = \frac{\sigma_{\mathrm{G}}k_{\mathrm{D}z}^{\mathrm{TM}}}{\omega\varepsilon_0\varepsilon_{\mathrm{D}}}$, and $\varsigma^{\mathrm{TE}} = -1$ together with $\varsigma^{\mathrm{TM}} = 1$. We can obtain the transmittance $T = |t|^2 = \left|\frac{1}{M_{\mathrm{PC}}(1,1)}\right|^2$ and the reflection $R = |r|^2 = \left|\frac{M_{\mathrm{PC}}(2,1)}{M_{\mathrm{PC}}(1,1)}\right|^2$. Besides, the reflection phase φ_r can be extracted from the reflection coefficient $r = |r|e^{i\varphi_r}$.

Subsequently, an extra metal layer E (like silver in the main text) is attached in front of the PC. Now the total transfer matrix can be expressed as $M_{\mathrm{Ag+PC}} = F_0^{-1}(F_{\mathrm{E}}P_{\mathrm{E}}F_{\mathrm{E}}^{-1})[(F_{\mathrm{C}}P_{\mathrm{C}}U_{\mathrm{G}}P_{\mathrm{D}}F_{\mathrm{D}}^{-1})^S F_{\mathrm{B}}P_{\mathrm{B}}F_{\mathrm{B}}^{-1}]^N T_0 = F_0^{-1}(F_{\mathrm{E}}P_{\mathrm{E}}F_{\mathrm{E}}^{-1})F_0 \cdot F_0^{-1}[(F_{\mathrm{C}}P_{\mathrm{C}}U_{\mathrm{G}}P_{\mathrm{D}}F_{\mathrm{D}}^{-1})^S F_{\mathrm{B}}P_{\mathrm{B}}F_{\mathrm{B}}^{-1}]^N F_0 = M_{\mathrm{Ag}} \cdot M_{\mathrm{PC}}$. In that case, we can divide the joint structure into two independent parts, and it is reasonable and convenient to discuss their properties separately. For the former part (the metal layer E), it is easy to obtain an analytical description

$$M_{\mathrm{Ag}} = F_0^{-1}(F_{\mathrm{E}}P_{\mathrm{E}}F_{\mathrm{E}}^{-1})F_0 = \begin{pmatrix} \cos(k_{\mathrm{E}z}d_{\mathrm{E}}) - \frac{i}{2}(\frac{N_0}{N_{\mathrm{E}}} + \frac{N_{\mathrm{E}}}{N_0})\sin(k_{\mathrm{E}z}d_{\mathrm{E}}) & \frac{i}{2}(\frac{N_0}{N_{\mathrm{E}}} - \frac{N_{\mathrm{E}}}{N_0})\sin(k_{\mathrm{E}z}d_{\mathrm{E}}) \\ \frac{i}{2}(-\frac{N_0}{N_{\mathrm{E}}} + \frac{N_{\mathrm{E}}}{N_0})\sin(k_{\mathrm{E}z}d_{\mathrm{E}}) & \cos(k_{\mathrm{E}z}d_{\mathrm{E}}) + \frac{i}{2}(\frac{N_0}{N_{\mathrm{E}}} + \frac{N_{\mathrm{E}}}{N_0})\sin(k_{\mathrm{E}z}d_{\mathrm{E}}) \end{pmatrix}. \quad \text{(S13)}$$

Noticing the conditions $\det(M_{\mathrm{Ag}}) = 1$ and $M_{\mathrm{Ag}}(2,1) = -M_{\mathrm{Ag}}(1,2)$, we can abstract this matrix as

$$M_{\mathrm{Ag}} = \begin{pmatrix} \mathcal{U} & -\mathcal{U}r_1 e^{i\varphi_{r1}} \\ \mathcal{U}r_1 e^{i\varphi_{r1}} & 1/\mathcal{U} - \mathcal{U}r_1^2 e^{i2\varphi_{r1}} \end{pmatrix}, \quad \text{(S14)}$$

where $r_{\mathrm{Ag}} = r_1 e^{i\varphi_{\mathrm{Ag}}} = r_1 e^{i\varphi_{r1}}$ is the reflection coefficient of the metal layer. Since we pay primary attention to the reflection phase in the following discussion, it is necessary to determine the phase of the element $\mathcal{U}$. Given that the working frequency is near the formant of silver, $|\mathrm{Re}(\varepsilon_{\mathrm{E}})| \gg |\mathrm{Im}(\varepsilon_{\mathrm{E}})|$ and $\mathrm{Re}(\varepsilon_{\mathrm{E}}) \ll 0$, hence $k_{\mathrm{E}z}^{\mathrm{TM}}, k_{\mathrm{E}z}^{\mathrm{TM}}, N_{\mathrm{E}} \in \mathbb{I}$ (the pure imaginary set), and $M_{\mathrm{Ag}}(2,1) = \mathcal{U}r_1 e^{i\varphi_{r1}} = \frac{i}{2}\left(-\frac{N_0}{N_{\mathrm{E}}} + \frac{N_{\mathrm{E}}}{N_0}\right)\sin(k_{\mathrm{E}z}d_{\mathrm{E}}) = |M_{\mathrm{Ag}}(2,1)|e^{i\varphi_{\mathrm{E}}} \to |M_{\mathrm{Ag}}(2,1)|e^{-i\pi/2}$. In that case, $\mathcal{U} = |\mathcal{U}|e^{i\varphi_{\mathcal{U}}} \to |\mathcal{U}|e^{-i(\pi/2+\varphi_{r1})}$. Considering the transmission coefficient $t = 1/\mathcal{U}$, the transmission phase of the layer E can also be determined as $\pi/2 + \varphi_{r1}$. For the latter part (the PC),

we abstract the matrix as $M_{\text{PC}} = \begin{pmatrix} \mathcal{V} & \cdots \\ \mathcal{V} r_2 e^{i\varphi_{r2}} & \cdots \end{pmatrix}$ directly due to its complex structure, where $r_{PC} = r_2 e^{i\varphi_{PC}} = r_2 e^{i\varphi_{r2}}$ is the reflection coefficient of the PC. Here since our main concerns are the forward transmittance and reflection, $M_{\text{PC}}(1,2)$ and $M_{\text{PC}}(2,2)$ are neglected temporarily. Finally, we can get the total matrix of the joint metal-PC structure

$$M_{\text{Ag+PC}} = \begin{pmatrix} \mathcal{U}\mathcal{V} - \mathcal{U}\mathcal{V} r_1 r_2 e^{i(\varphi_{r1}+\varphi_{r2})} & \cdots \\ \mathcal{U}\mathcal{V} r_1 e^{i\varphi_{r1}} + \mathcal{V} r_2 e^{i\varphi_{r2}}/\mathcal{U} - \mathcal{U}\mathcal{V} r_1^2 r_2 e^{i(2\varphi_{r1}+\varphi_{r2})} & \cdots \end{pmatrix}, \tag{S15}$$

and the reflection is

$$r_{\text{Ag+PC}} = \frac{M_{\text{Ag+PC}}(2,1)}{M_{\text{Ag+PC}}(1,1)} = r_1 e^{i\varphi_{r1}} + \frac{r_2 e^{i\varphi_{r2}}}{\mathcal{U}^2 - \mathcal{U}^2 r_1 r_2 e^{i(\varphi_{r1}+\varphi_{r2})}}, \tag{S16}$$

where two terms correspond to the contributions of two reflection channels based on the silver and the PC, respectively. Noticing that the variables $\mathcal{U}$, r_1 and φ_{r1} can be described analytically through Eq. (S13), and $r_1, r_2 \to 1$ in the discussed frequency of band gaps. Therefore, we can only use the extracted reflection phase φ_{r2} to explain some phenomena of the complex composite construction.

In Ref. [S6], a stable surface state should satisfy the condition of $\varphi_{r1} + \varphi_{r2} = 2n\pi$ $(n \in \mathbb{Z})$. Without loss of generality, we set $\varphi_{r1}, \varphi_{r1} \in [-\pi, \pi]$, and $n = 0$. Now we survey this condition again through the above method. A critical factor is the coefficient $\frac{1}{\mathcal{U}^2 - \mathcal{U}^2 r_1 r_2 e^{i(\varphi_{r1}+\varphi_{r2})}}$, which can be seen as the influence to the PC after the incident wave propagations through the metal layer E. Combining with $\mathcal{U} = |\mathcal{U}| e^{i\varphi_{\mathcal{U}}} \to |\mathcal{U}| e^{-i(\pi/2+\varphi_{r1})}$, $T_{\text{Ag}} = 1/|\mathcal{U}|^2$, $R_{\text{Ag}} = r_1^2$, and $r_1 \sim r_2 \to 1$, we can get $\frac{1}{\mathcal{U}^2 - \mathcal{U}^2 r_1 r_2 e^{i(\varphi_{r1}+\varphi_{r2})}} \to \frac{T_{\text{Ag}}}{1 - R_{\text{Ag}} e^{i(\varphi_{r1}+\varphi_{r2})}} e^{i(\pi+2\varphi_{r1})}$. When $\varphi_{r1} + \varphi_{r2} = 2n\pi$, equation (S16) can be approximatively simplified as $r_{\text{Ag+PC}} = r_1 e^{i\varphi_{r1}} + \frac{r_2 e^{i\varphi_{r2}}}{\mathcal{U}^2 - \mathcal{U}^2 r_1 r_2 e^{i(\varphi_{r1}+\varphi_{r2})}} \to$

$r_1 e^{i\varphi_{r1}} + \frac{T_{\mathrm{Ag}}}{1-R_{\mathrm{Ag}} e^{i(\varphi_{r1}+\varphi_{r2})}} e^{i(\pi+2\varphi_{r1})} r_2 e^{i\varphi_{r2}} = r_1 e^{i\varphi_{r1}} + \frac{T_{\mathrm{Ag}}}{1-R_{\mathrm{Ag}} e^{i(\varphi_{r1}+\varphi_{r2})}} r_2 e^{i(\pi+2\varphi_{r1}+\varphi_{r2})} \xrightarrow{\varphi_{r1}+\varphi_{r2}=0} r_1 e^{i\varphi_{r1}} + r_2 e^{i(\pi+\varphi_{r1})} \to 0$. It can be seen the whole reflection channel closes due to the destructive interference. For $\varphi_{r1} + \varphi_{r2} \to 0^+$ or 0^-, these two terms are not collinear and reverse in the complex plane any longer. At present, the reflection $R_{\mathrm{Ag+PC}} = |r_{\mathrm{Ag+PC}}|^2 > 0$. Therefore, the condition $\varphi_{r1} + \varphi_{r2} = 2n\pi$ $(n \in \mathbb{Z})$ can identify surface states.

Then we illustrate the above condition in the actual structure for the TM polarization as the supplement of Fig. 3. As is shown in Fig. S5(a), there also exist two kinds of photonic insulators at the same band gap, respectively corresponding to ENG ($\varphi_r \in [0, \pi]$) and MNG ($\varphi_r \in [-\pi, 0]$) phases (just opposite to those for the TE polarization), which are separated by a white line ($\varphi_r = 0$) [S7]. As an example, the detailed information of $k_\rho/k_0 = 0.4$ and $k_\rho/k_0 = 0.8$ is plotted respectively in Figs. S5(b) and S5(c), belonging to the different regions inside and outside the nodal ring. When we cut a period $[-\pi, \pi]$ of the axis φ_r, twist and glue end to end, the plane of $f - \varphi_r$ is molded into a cylinder. On the cylinder, the upper region (colored by gradient blue) corresponds to the ENG phase, while the lower region (colored by gradient red) corresponds to the MNG phase. The boundary of $\varphi_r = \pi$ and the center of $\varphi_r = -\pi$ are highlighted by black and white dotted lines, respectively. There is no doubt the metal layer E (the red line) corresponds to the ENG phase. And for $k_\rho/k_0 = 0.4$, the condition $\varphi_{r1} + \varphi_{r2} = 0$ is satisfied near the frequency $f = 286.818$ THz (marked by the cyan star), corresponding to a dip of the reflection $R_{\mathrm{Ag+PC}}$ (the purple line) in Fig. S5(b). Similarly, for $k_\rho/k_0 = 0.8$, the corresponding frequency change into $f =$

288.835 THz in Fig. S5(c).

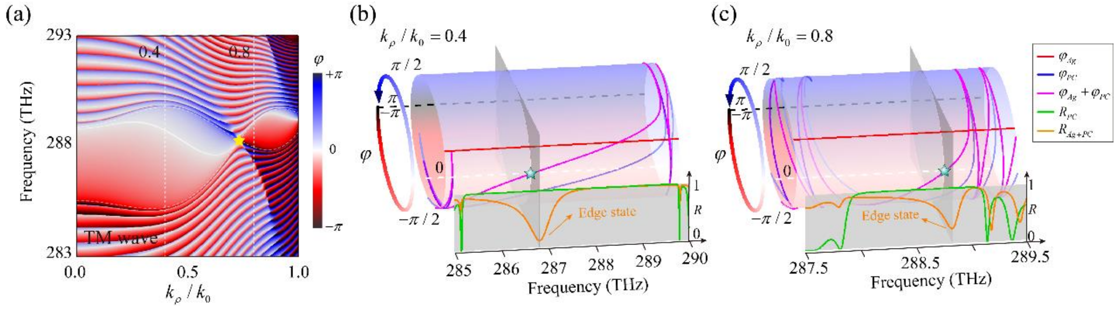

FIG. S5. (a) Phase diagrams φ_r of the PC [(CGD)22B]20 for the TM wave. When (b) $k_\rho/k_0 = 0.4$ and (c) $k_\rho/k_0 = 0.8$, the calculated reflection phase φ_{Ag} (red), φ_{PC} (blue) together with $\varphi_{\mathrm{Ag}} + \varphi_{\mathrm{PC}}$ (magenta) is illustrated on the cylinder, and related reflection R_{PC} (green) together with $R_{\mathrm{Ag+PC}}$ (brown) is illustrated on the vertical rectangular window. On the cylinder, the upper region (colored by gradient blue) corresponds to the ENG phase, while the lower region (colored by gradient red) corresponds to the MNG phase, where the boundary of $\varphi_r = \pi(-\pi)$ and the center of $\varphi_r = 0$ are highlighted by black and white dotted lines, respectively. The points satisfied $\varphi_{\mathrm{Ag}} + \varphi_{\mathrm{PC}} = 0$ are marked by cyan stars.

In that case, a pair of edge states is supported inside and outside the Weyl nodal ring (WNR), corresponding to the bilateral DSSs. As a comparison, we modify the unit cell of PC from the form of (AB) to $(\mathrm{B}''\mathrm{AB}'')$ to restore the inversion symmetry. Here $d_{\mathrm{B}''} = 0.5 d_{\mathrm{B}}$, and other parameters remain unchanged with the structure $(\mathrm{AB})_{20}$ in Fig. 2(a). As is shown in Fig. S6(a), the bandgap region for PC, inside or outside the WNR, is filled with only one component, ENG (gradient red) or MNG (gradient blue), respectively. Hence, the condition $\varphi_{\mathrm{Ag}} + \varphi_{\mathrm{PC}} = 0$ is only met outside the WNR in Figs. S6(b)–S6(c), leading to a unilateral DSS ultimately in Fig. S6(d). Similar situations can be found for the TM wave in Figs. S6(e)–S6(h). At this moment, it comes back to the case discussed in Ref. [S6]. It follows that the above semi-analytic method can provide service for appreciating the inherent mechanism of the surface state condition. In the next section, we will introduce more theoretical applications about this method.

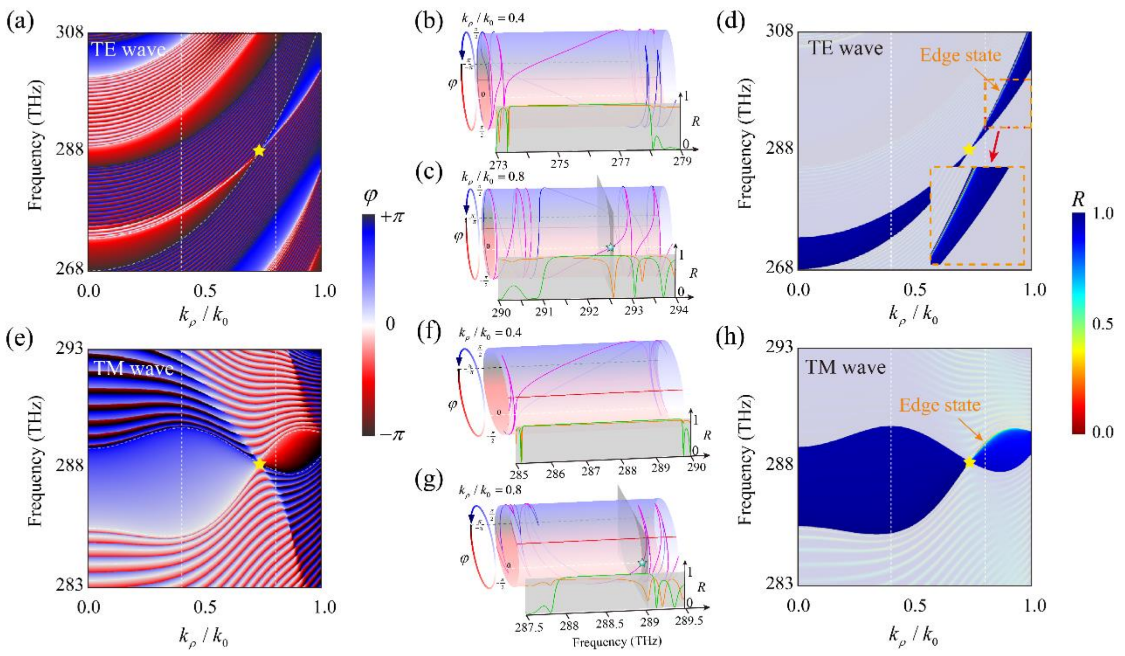

FIG. S6. Unilateral DSSs in double WNRs. (a) Phase diagram φ_r of the PC $(\mathrm{B''AB''})_{20}$ with inversion symmetry for the TE wave. Compared with the structure $(\mathrm{AB})_{20}$ in Fig. 2(a), $d_{\mathrm{B''}} = 0.5d_{\mathrm{B}}$ here, and other parameters remain unchanged. (b) When $k_\rho/k_0 = 0.4$, the reflection phases φ_{Ag} for the silver layer E (red), φ_{PC} for the PC (blue) together with their sum $\varphi_{\mathrm{Ag}} + \varphi_{\mathrm{PC}}$ (magenta) are illustrated on the cylinders, and related reflections R_{PC} (green) together with $R_{\mathrm{Ag+PC}}$ (brown) are illustrated in the insets. The boundary of $\varphi_r = \pi(-\pi)$ and the center of $\varphi_r = 0$ are highlighted by black and white dotted lines, respectively. (c) Similar to (b), but for $k_\rho/k_0 = 0.8$. The unique point satisfied $\varphi_{\mathrm{Ag}} + \varphi_{\mathrm{PC}} = 0$ is marked by cyan stars. (d) Reflection spectra of the PC $(\mathrm{B''AB''})_{20}$ for the TE wave. The rest is shaded except the interested gap, and the inset is an enlarged view. DSS only exists in the region outside the WNR. (e)–(h) Similar to (a)–(d), but for the TM wave.

Sec. V. Singularities pairs and degenerate bound states in the continuum

In this section, we inspect the relevant evolution of singularities from two different aspects, the winding of reflection phases and the space of complex frequency. As is shown in Fig. S7(a), we take the singularity $\mathcal{A}$ for the TE mode in Fig. 3(f) as an example. Without loss of generality, we trace an anticlockwise rectangle closed loop $\mathcal{A}_1\mathcal{A}_2\mathcal{A}_3\mathcal{A}_4\mathcal{A}_1$, which is defined by the lines $\theta_1 = 51.5°$ ($k_\rho/k_0 = 0.7826$, the red line), $f_1 = 291\mathrm{THz}$ (the purple line), $\theta_2 = 52°$($k_\rho/k_0 = 0.7880$, the blue line) and $f_2 = 293\mathrm{THz}$ (the cyan line). In view of the utility and universality of the practical

applications, the wave vector k_ρ is substituted with the incident angle θ here. Since the reflection phase almost remains unchanged along the horizontal routes $\mathcal{A}_2\mathcal{A}_3$ and $\mathcal{A}_4\mathcal{A}_1$, we can obtain the topological charge $\upsilon = \left(\frac{1}{2\pi}\right)\oint_{\mathcal{A}_1\mathcal{A}_2\mathcal{A}_3\mathcal{A}_4\mathcal{A}_1} d\varphi_r = W(\theta_1) + W(\theta_2)$, where φ_r is the reflection phase of the structure Ag + PC, and W is the winding number of the corresponding angle. As is shown in Fig. S7(b), there exists a +1 charge in the discussed region $\mathcal{A}$. And more incident angles are illustrated in Fig. S7(c). For smaller angles like $\theta = 51.5°$ and $\theta = 51.7°$, there are obviously falling edges. With the increase of the angle, the linewidth of the falling edge reduces to zero gradually and forms a Heaviside-like phase jump, where completes winding ultimately and corresponds to a singularity. Therefore, the trend of the reflection phases can determine the positions of nontrivial singularities through simply changing incident angles. Meanwhile, a giant phase change exists for a wide range of the incident angles, almost from $\theta \in [52°, 60°]$. Additionally, the situation near the point $\mathcal{B}$ is on the contrary in Fig. S7(e), which corresponds to a -1 charge.

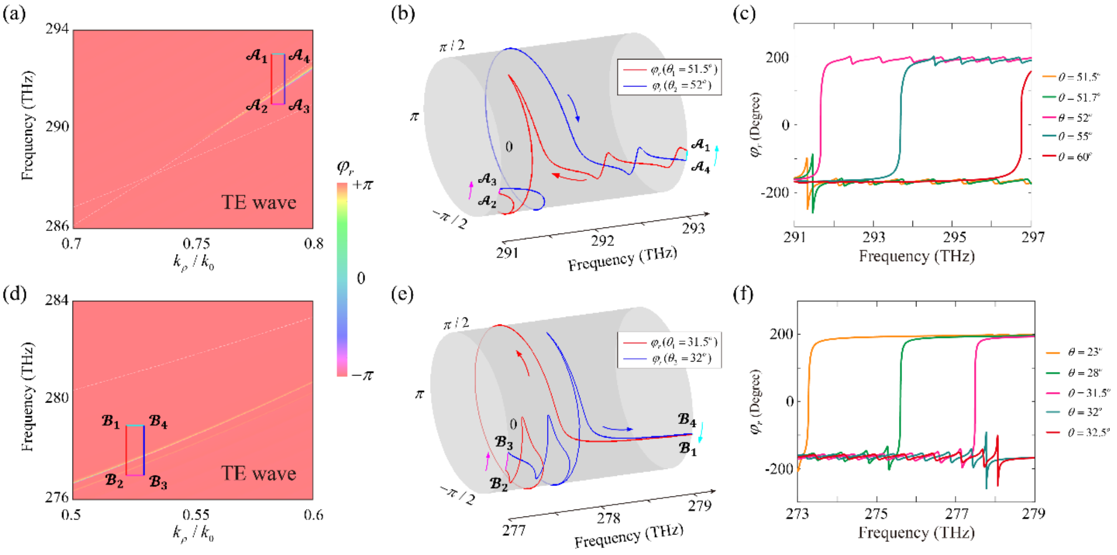

FIG. S7. Phase diagrams φ_r of the composite structure E[(CGD)$_{22}$B]$_{20}$ for the TE wave. (a) The region near the singularity $\mathcal{A}$ is surrounded by an anticlockwise rectangle closed loop

$\mathcal{A}_1\mathcal{A}_2\mathcal{A}_3\mathcal{A}_4\mathcal{A}_1$. (b) Reflection phase variation with topological charge (winding number) +1 when tracing the loop on the cylinder, and (c) similar variation with more fixed incident angles near the singularities $\mathcal{A}$. (d)–(f) Similar to (a)–(c), but around the singularity $\mathcal{B}$ with topological charge (winding number) -1.

On the other hand, it can be predicted that when two singularities with different topological charges merge in the momentum space, the total charge will become zero due to charge conservation [S8]. Here such a phenomenon is observed in the space of complex frequency. Out of completeness, we take both TE and TM polarizations as an example. According to Eq. (S15), the reflection zero condition can be described as:

$$\mathcal{U}r_1e^{i\varphi_{r1}} + r_2e^{i\varphi_{r2}}/\mathcal{U} - \mathcal{U}r_1^2r_2e^{i(2\varphi_{r1}+\varphi_{r2})} = 0. \tag{S17}$$

Figure S8(a) shows the dispersion of the reflection-zeros in $f_r - k_\rho$ plane, while figure S8(b) in $f_i - k_\rho$ plane for the TE wave, corresponding to figure 4 in the main text. Here f_r (f_i) represents the real (imaginary) part of the solution. For *N*=20, the dispersion intersects with the axis of $f_i = 0$, which symbolizes pure real excitation frequency of the surface states, near the points $k_\rho/k_0 = 0.5292$ and $k_\rho/k_0 = 0.7849$, corresponding to the singularities $\mathcal{B}$ and $\mathcal{A}$ in Fig. 3(f). When increasing the number of the unit cell (*N*=200), the leakages of radiation reduce, and the singularity pair merges near the degenerate point ($k_\rho/k_0 = 0.73$), which forms the bound state in the continuum. Similar conclusions can be obtained for the TM mode at the same point. For *N*=20, the dispersion intersects with the axis of $f_i = 0$ near the points $k_\rho/k_0 = 0.6945$ and $k_\rho/k_0 = 0.7539$, corresponding to the singularities $\mathcal{D}$ and $\mathcal{C}$ in Fig. 3(g). Each above singularity pair comes from the same nodal line corresponding to the ultimate BIC. In view of the properties of quasi-DNLSs, these BICs with different polarizations tend to degenerate in the $f - k_\rho$ space. In addition, we notice another

singularity $\mathcal{E}$ with -1 charge near the points $k_\rho/k_0 = 0.8711$, which comes from the neighbor nodal line for the TE wave. On the other hand, the scattering matrix can be described as

$$\mathcal{S} = \begin{pmatrix} M(2,1)/M(1,1) & [M(1,1)M(2,2) - M(1,2)/M(2,1)]/M(1,1) \\ 1/M(1,1) & -M(1,2)/M(1,1) \end{pmatrix}. \quad \text{(S18)}$$

Except the reflection zero point, there is another special point, the pole point, which takes the form of

$$M_{\text{Ag+PC}}(1,1) = \mathcal{UV} - \mathcal{UV} r_1 r_2 e^{i(\varphi_{r1}+\varphi_{r2})} = 0. \quad \text{(S19)}$$

Interestingly, the bound state in the continuum satisfies the condition of both reflection zero points and pole points at the same time, which can be related to the classical model in one-port systems [S8]. Similar situations appear in the structures of isolated WNLSs and DNLSs in Figs. S9 and S10, respectively. Therefore, DSSs become an intriguing bridge between Weyl semimetals and BICs.

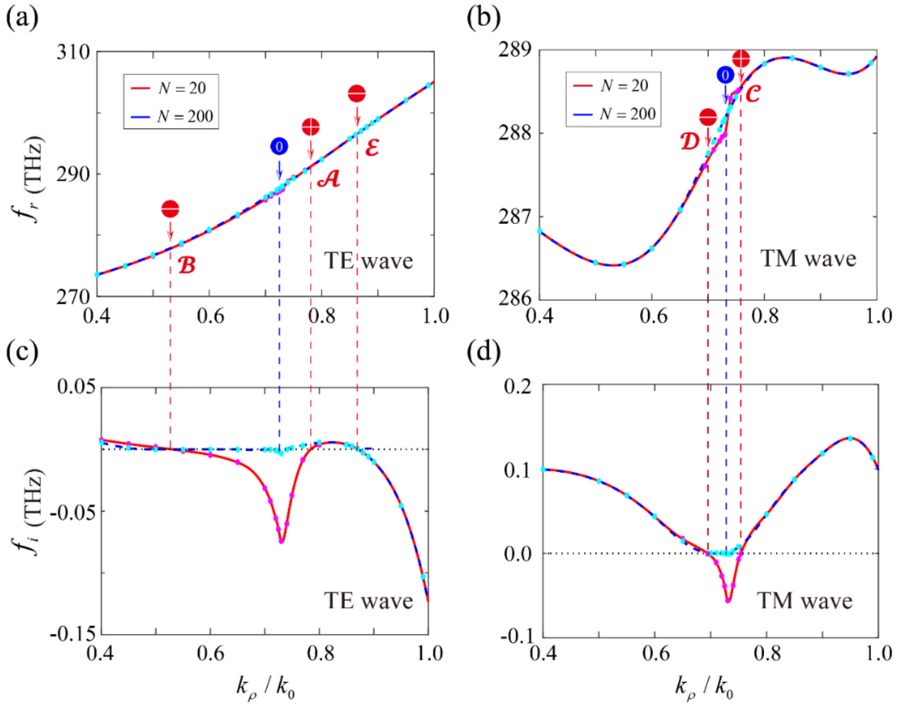

FIG. S8. Reflection-zero dispersion for bilateral DSSs of quasi-DNLS in the real frequency space [(a), (c)], and imaginary frequency space [(b), (d)] for TE and TM waves. The positions of singularities are indicated by red arrows, while those of BICs are indicated by blue arrows.

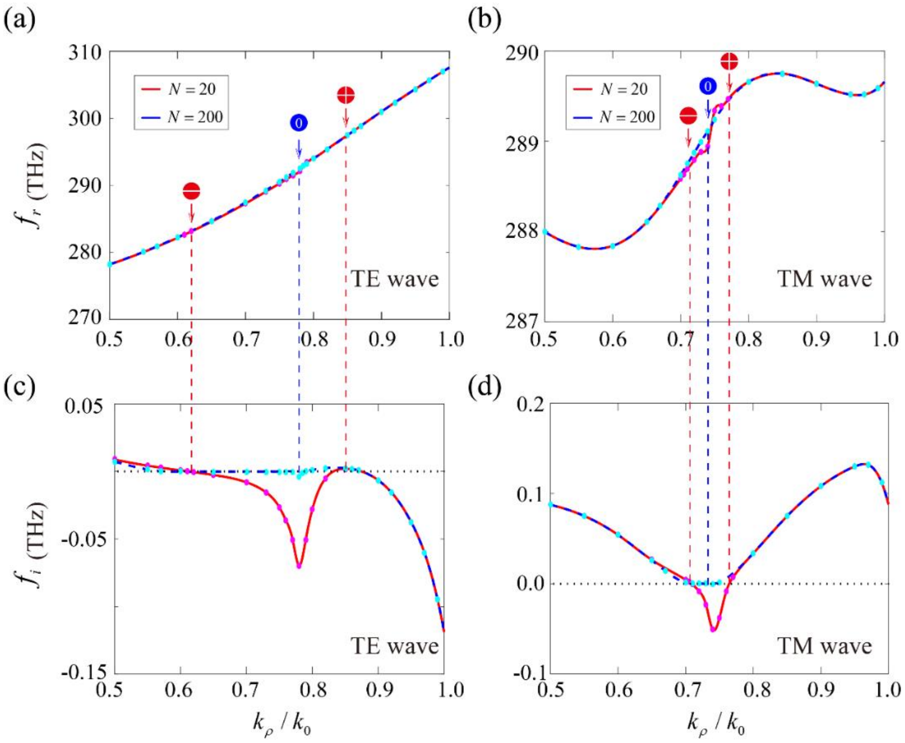

FIG. S9. Reflection-zero dispersion for bilateral DSSs of isolated WNLSs in the (a), (c) real frequency space, and (b), (d) imaginary frequency space for TE and TM waves. The positions of singularities are indicated by red arrows, while those of BICs are indicated by blue arrows.

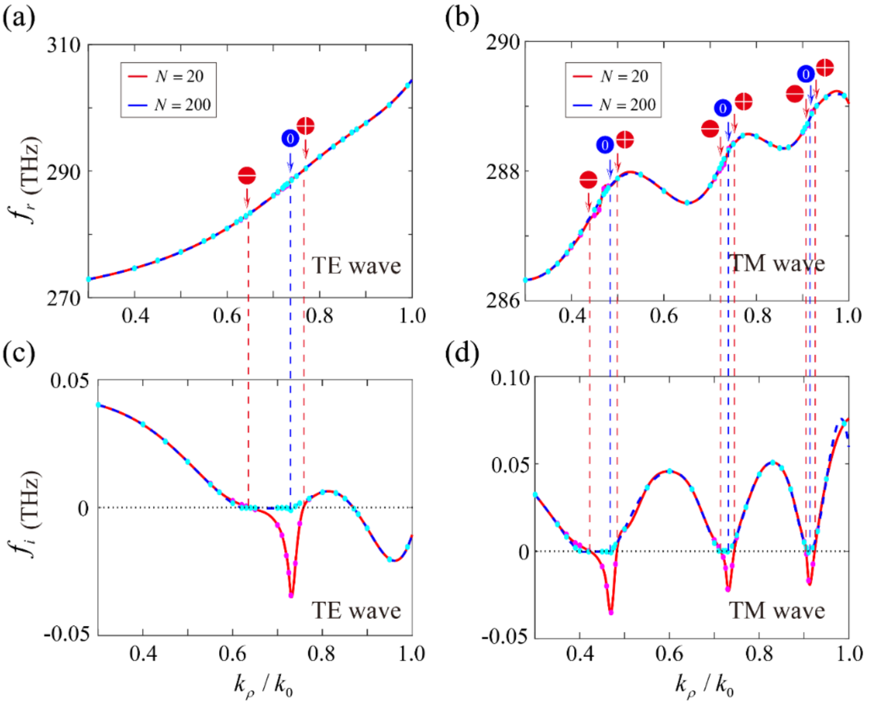

FIG. S10. Reflection-zero dispersion for bilateral DSSs of DNLSs in the (a), (c) real frequency space, and (b), (d) imaginary frequency space for TE and TM waves. The positions of singularities are indicated by red arrows, while those of BICs are indicated by blue arrows.

To explore the properties near the BIC, we further observe the reflection with fixed

k_ρ/k_0 in Fig. S11. Take DNLS in Fig. S11(d) as an example, the linewidth of the edge state is restricted by the band edge. Therefore, it becomes narrow and narrow when coming close to the WNR (along the direction of the arrow), and vanishes in the bulk states ultimately at $k_\rho/k_0 = 0.7355$ for the WNR (yellow star), which corresponds to BIC. Similar situations can also be found in the phase of quasi-DNLS [Fig. S11(e)] and isolated WNLS [Fig. S11(f)]. Note that the DOF of translation is endowed to BIC from the decoupled multi-quasiparticle phase quasi-DNLS [Fig. S11(e)] to isolated WNLSs [Fig. S11(f)], which may give rise to a freely controllable BIC.

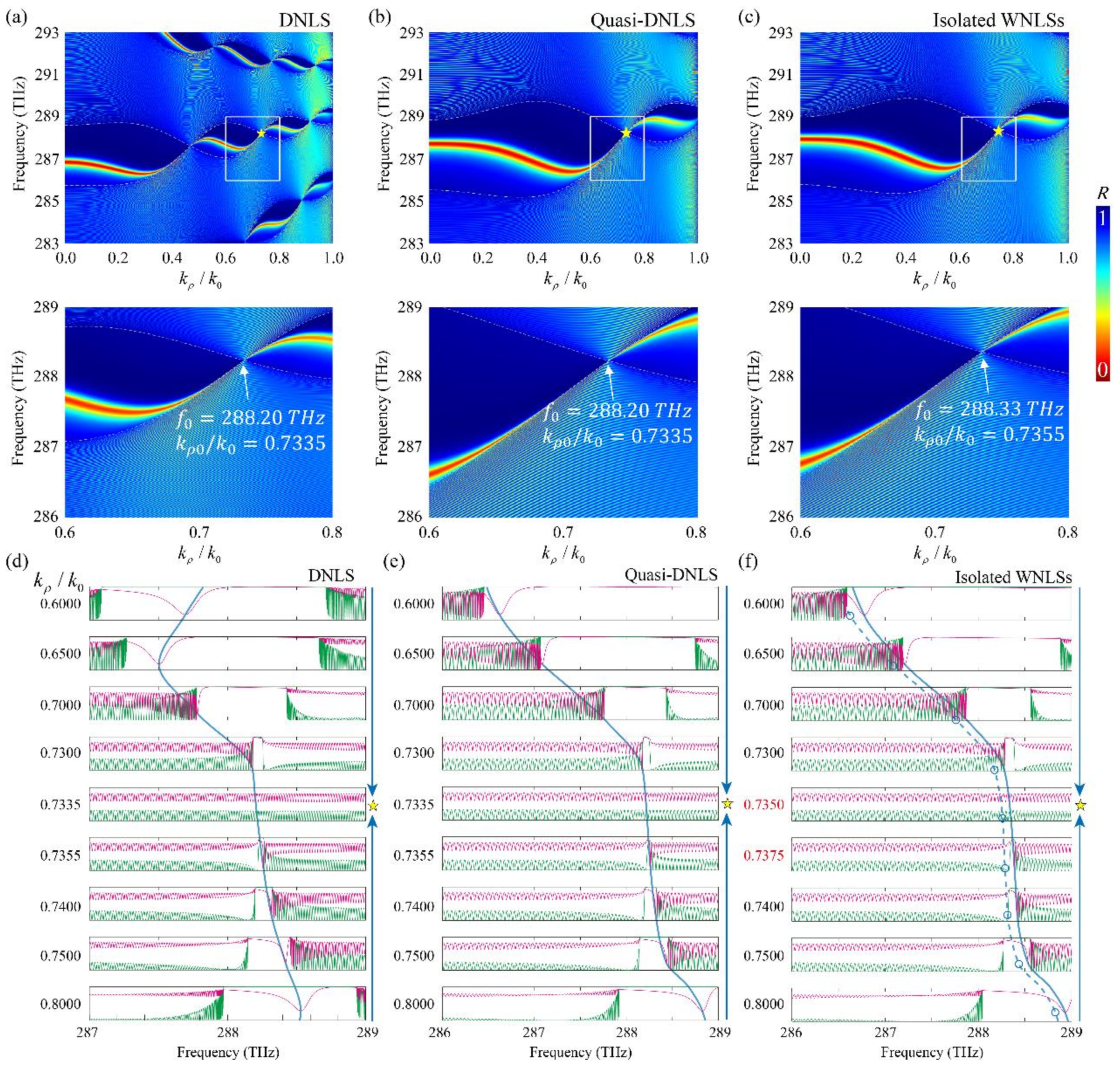

FIG. S11. Reflection diagrams of bilateral DSSs for the phase of (a) DNLS [(CGD)$_{44}$B']$_N$ with

$E_F = 0.93$ eV, (b) quasi-DNLS $[(CGD)_{22}B]_N$ with $E_F = 0.93$ eV, and (c) isolated WNLSs $[(CGD)_{22}B]_N$ with $E_F = 1.43$ eV. And the unit cell number is N=200. The second row is an enlarged view of the white box in the first row. Reflection $\varphi_{\rm PC}$ for the PC (green lines) and the hyper-crystal (purple lines) with several fixed k_ρ/k_0 for the phase of (d) DNLS, (e) quasi-DNLS, and (f) isolated WNLSs. The moments of WNR are marked by yellow stars, and the bilateral DSSs are represented by blue lines. As a comparison, the bilateral DSS of quasi-DNLS is also marked as the blue dashed line in (f).

Sec. VI. Berry curvature vortex near the nodal lines

In this section, we observe the Berry curvature distributions around the nodal lines as a supplement of the above discussions about topological properties.

At first, combining the Bloch boundary condition

$$\begin{pmatrix} H_{\rm in}^{+} \\ H_{\rm in}^{-} \end{pmatrix} = e^{-ik_z\Lambda} \begin{pmatrix} H_{\rm out}^{+} \\ H_{\rm out}^{-} \end{pmatrix}, \tag{S20}$$

with Eq. (S12), we can obtain the total wave equation:

$$\left[M_{\rm PC}(k_\rho) - e^{-ik_z\Lambda}\right] \begin{pmatrix} H_{\rm out}^{+} \\ H_{\rm out}^{-} \end{pmatrix} = 0. \tag{S21}$$

Through representation transformations [S6], the eigenvectors corresponding to the effective Hamiltonian of PC with structure in Fig. 2(a) can be expressed as $|u(\vec{k})\rangle = \begin{pmatrix} -iH_{\rm out}^{+} \\ iH_{\rm out}^{-} \end{pmatrix}$. And the Berry curvature can be calculated by $\Omega = i\nabla_{\vec{k}} \times \langle u(\vec{k})|\nabla_{\vec{k}}|u(\vec{k})\rangle$ [S8]. Without loss of generality, we take the TM mode as an example. As shown in Figs. S12(a) and S12(b), the Berry curvature possesses a configuration of THE vortex and is concentrated near the position of nodal lines like the results in Ref. [S9]. Similarly, we can get the Berry curvature distributions of bilateral DSSs in Figs. S12(c) and S12(d). It is noteworthy that Euclidean norms of the Berry curvature increase significantly, which may owe to the potential BIC.

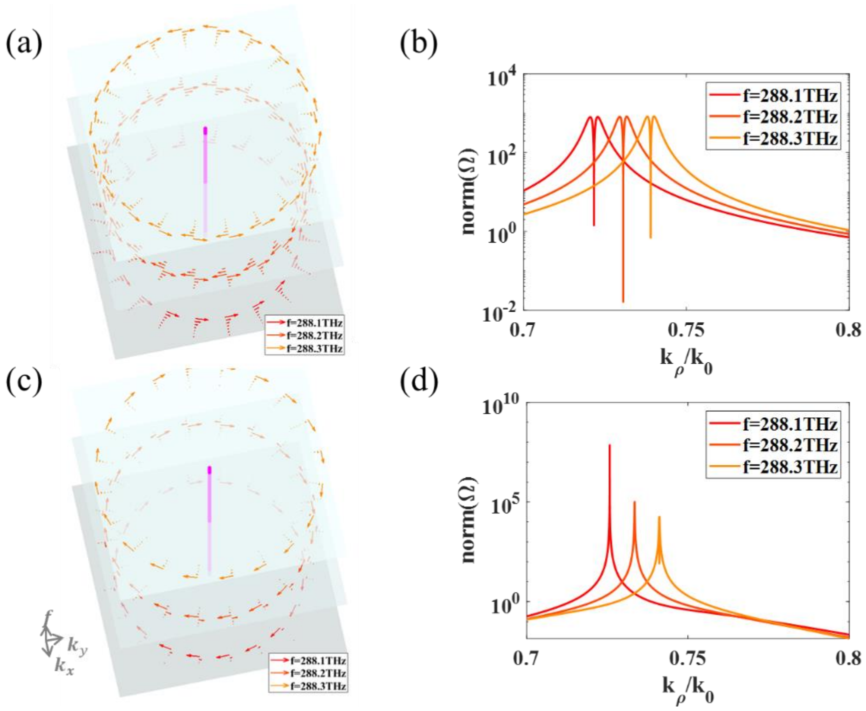

FIG. S12. The Berry curvature distributions of the structures (a) $[(CGD)_{22}B]_N$ and (c) $E[(CGD)_{22}B]_{20}$ on the $k_z = 0$ plane with $k_\rho/k_0 \in [0.725, 0.740]$. The original points of $\vec{k}$ are marked by the purple line. (b), (d) Corresponding Euclidean norms of the Berry curvature calculated along k_ρ/k_0.

Sec. VII. Strategy for dual-mode sensing

As is mentioned above, the energy leakage can split the BIC (pinned near the nodal line) into a singularity pair, which is characterized by Heaviside-like phase jumps in Figs. S7(c) and S7(f). In fact, such dramatical phase jumps are quite sensitive to environmental disturbances, such as the external refractive indexes [S10, S11], molecule attachment [S12, S13], and temperature [S14], which lays a solid foundation for sensors with ultrahigh sensitivities. Most of these schemes employ spectroscopic ellipsometry methods, which are based on the amplitude and phase differences between TE (s) and TM (p) waves [S10]. Despite the elliptically polarized light have two orthogonal polarizations, TE and TM components tend to be non-decoupling in previous works, corresponding to a single mode. Meanwhile, the presence of

singularities mainly depends on the TM component, while the TE component of the elliptically polarized light can hardly contribute to the process of sensing (the former corresponds to the zero point of the complex reflection ratio $\rho = \frac{r^{\text{TM}}}{r^{\text{TE}}}$, while the latter corresponds to the pole point). To improve the scheme in this respect, a new strategy is necessary. We notice singularity pairs for TE and TM waves possess several similarities as well as differences, which form a novel dual mode. In the main text, a joint 2D colormap is employed to reveal properties of the dual mode in Fig. 3(i). Compared with the classical single mode, like a separate line in Fig. 3(h), such dual mode seems to link with a more extensive space, which is able to support more states of system. To some extent, this property may infuse new blood into conventional schemes of singularity applications. In this section, we make an attempt to provide one of strategies for similar dual-mode sensing.

To exhibit more unique characteristics of 2D material in our structure, the ion concentration in electrolyte is selected as the environmental disturbance, which can alter the Fermi energy E_F of the graphene [S15]. And the following conclusions can be generalized to other disturbances easily. As is shown in Fig. S13(a), we envision the composite structure $E[(CGD)_{22}B]_{20}$ immersed in the electrolyte as a concentration sensor. The IR source launch the incident electromagnetic wave with two modes near the singularities $\mathcal{B}$ and $\mathcal{D}$. Mode 1: $\theta = 31°$ and $f = 277.2$ THz for the TE wave. Mode 2: $\theta = 43.9°$ and $f = 287.6$ THz for the TM wave. For applications, it is always troublesome to provide two modes with different incident angles and frequencies. Fortunately, the structure of the sensor can be further optimized based on

the flexible modulations of the double WNLSs system. The phase jumps are illustrated in Fig. S13(b) and S13(c) respectively. And the responses of reflection phases with the change of E_F under two modes are plotted in Fig. S13(d). The sensitivities of both modes are all close to the order of 10^4 deg/RIU. Since the change of ion concentration with the order of $10^{-3}\ \mathrm{mol \cdot L^{-1}}$ leads to the response of E_F with the order of 10^2 meV, the actual sensitivities may be higher. Moreover, a common issue of similar sensor based on singularities is the rigorous precision of incident angle. The sensitivities reduce rapidly with the deviation of incident angle. For singularities supported by surface states in WNLSs, such dramatical phase jumps exist for a wide range of the incident angles, as is shown in Figs. S7(c) and S7(f), which may owe to the properties of the surface states. Therefore, such composite structure may be one of the solutions to the issue. Noteworthy, the center working frequency shift with the change of the incident angle, which is connected to the dispersion of surface states. This may be conducive to another sensing scheme of incident angle, but also give a challenge to the calibration after preparation at the same time.

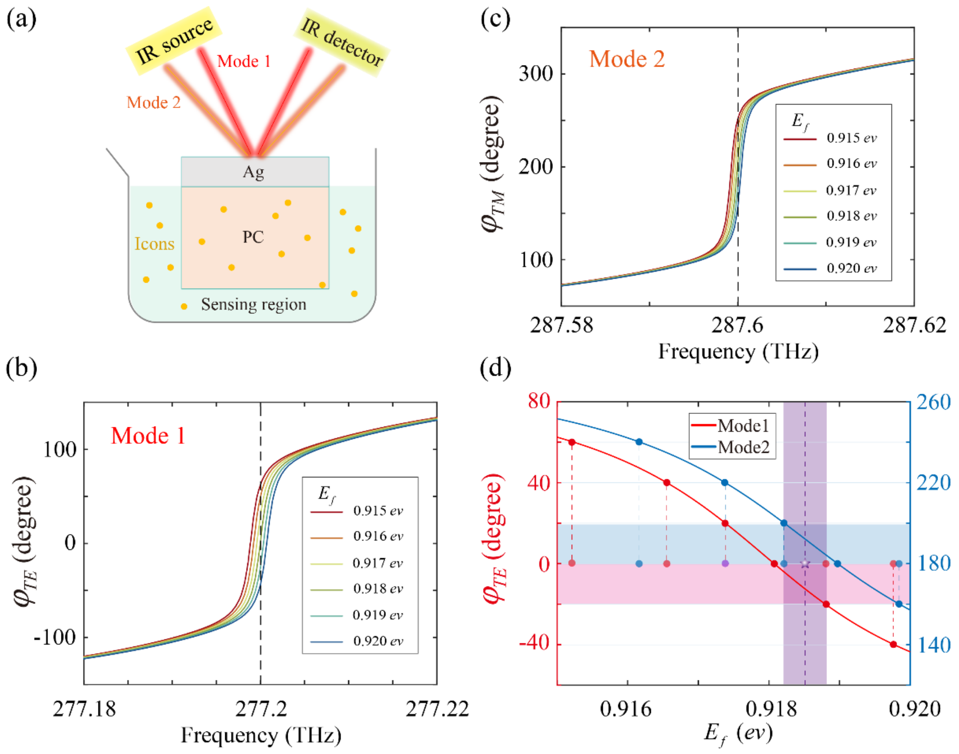

FIG. S13. A sensing strategy of dual-modes. (a) Sensing scheme based on phase singularities. And reflection phase as the frequency is changed near (b) Mode 1 and (c) Mode 2. (d) The responses of reflection phases with the change of E_F under Mode 1 (red) and Mode 2 (blue).

On the other hand, conventional sensing strategies based on a single singularity are facing challenges in recent years. Especially, the precise reading of reflection phases is limited by the fundamental resolution limit and other noise-induced bounds in practical applications [S16]. To reveal the potential advantages of this strategy clearly, we assume a wide and fixed confidence interval of response $\varphi_r = 20°$. Take the case $E_F = 0.9185$ eV as an example, which is marked by the purple star in Fig. S13(d). Under Mode 1, the response region (light red block) is $\varphi_r \in [-20°, 0°]$, corresponding to $E_F \in [0.91807, 0.91880]$ eV. Under Mode 2, the response region (light blue block) is $\varphi_r \in [180°, 200°]$, corresponding to $E_F \in [0.91822, 0.91897]$ eV. Taking both modes into account, we can confirm $E_F \in [0.91822, 0.91880]$ eV ultimately, and the precision improves. Then we can project the special nodes (red or blue) on the response

curves, which is linked with the confidence interval, to the axis of environment variable E_F. For a fixed region of the environment variable, we take $\mathbb{P}(\text{mode})$ to describe the set of these nodes under corresponding mode, and $\eta[\mathbb{P}(\text{mode})]$ to describe the element number of the set. It is expected the average confidence interval of the environment variable with the dual-mode strategy is proportional to $1/\{\eta[\mathbb{P}(\text{Mode 1}) \cup \mathbb{P}(\text{Mode 2})] + 1\}$, while those with the single-mode strategy are proportional to $1/\{\eta[\mathbb{P}(\text{Mode 1})] + 1\}$ or $1/\{\eta[\mathbb{P}(\text{Mode 2})] + 1\}$, respectively. In Fig. S13(d), $\eta[\mathbb{P}(\text{Mode 1})] = 6$, $\eta[\mathbb{P}(\text{Mode 2})] = 5$, and $1/\eta[\mathbb{P}(\text{Mode 1}) \cup \mathbb{P}(\text{Mode 2})] = 10$. Here two points for different modes coincide on the axis near $E_F = 0.91378$ eV, which is marked by the purple point. In one word, such dual mode links with a more extensive space, which is able to support more states of the system, and the designed structure support two times of independent measurements simultaneously. This property may infuse new blood into conventional schemes of singularity applications.

Sec. VIII. Absorption losses and annihilation of singularities

In the above discussion, we ignore the losses of the metal layer D. In this section, we regard the losses as a kind of disturbance, and review parts of the conclusions from WNLSs to singularities. As is shown in Figs. S14(a)–S14(d), we plot the transmittance spectra as well as reflection spectra of the structure $[(CGD)_{22}B]_{20}$ for TE and TM waves. Due to the absorption losses of metal, both the transmittance in conduction bands and the reflection in forbidden bands reduce obviously, especially for large incident angles. This is probably related to the decay of evanescent waves in the propagation direction.

However, the linear dispersion of quasi-DNLSs still exists near the degenerate points. Similarly, figures S14(e) and S14(f) illustrate the surface states in the lossy case. We also observe the singularities in the space of complex frequency. Compared with the lossless case in Fig. S8, the reflection-zero dispersions almost remain unchanged in the real-frequency space (the top), while they shift down along the imaginary axis (the bottom), especially for the peak on the right of the nodal lines.

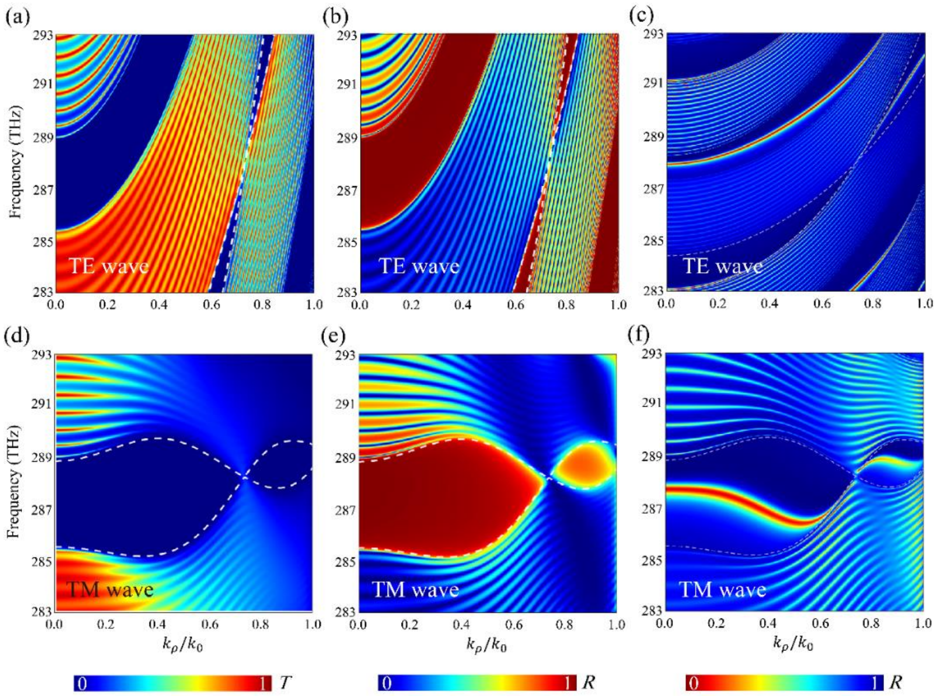

FIG. S14. (a) and (d) Transmittance spectra as well as (b) and (e) reflection spectra of the structure $[(CGD)_{22}B]_{20}$ for TE and TM waves. (c) and (f) Reflection spectra of the structure $E[(CGD)_{22}B]_{20}$ in the lossy case for TE and TM waves.

We notice another singularity $\mathcal{F}$ with -1 charge near the points $k_\rho/k_0 = 0.9804$, which comes from the neighbor nodal line for the TM wave, just like the singularity $\mathcal{E}$ in Figs. S8(a) and S8(b). At the same time, the singularities $\mathcal{A}$ and $\mathcal{E}$ with opposite charges disappear in the lossy case. By coincidence, the situations for TM and TE waves can represent two important steps in the evolutionary process of singularities. With absorption losses increasing, the peak above the axis of $f_i = 0$ drop gradually. And

two intersecting points (singularities), which come from two neighbor nodal lines respectively, approach each other, as is shown in Fig. S15(d). Ultimately, the peak is below the axis, and there are no intersecting points any longer. And two singularities with opposite charges annihilate each other, like $\mathcal{A}$ with +1 charge as well as $\mathcal{E}$ with -1 charge in Figs. S8(c) and S15(c). On the other hand, we focus on the region near the nodal lines. The influence of absorption losses is far stronger than that of radiation leakage, and simply increasing the number of the unit cell from 20 to 200 is unable to let the singularity pairs merge again in the lossy case. That is the losses in the HMMs may have destructive interferences to the degenerate BIC. Such optical losses can be compensated by gain media [S17] or reduced by thermal annealing [S18]. On the other hand, the above conclusions also refer to all-dielectric PCs. For example, we can replace the layer of HMMs with uniaxial materials, which can also reduce the difficulties in preparation. It should be noted that introducing of HMMs is only to reinforce the DOF of rotation (Sec. II).

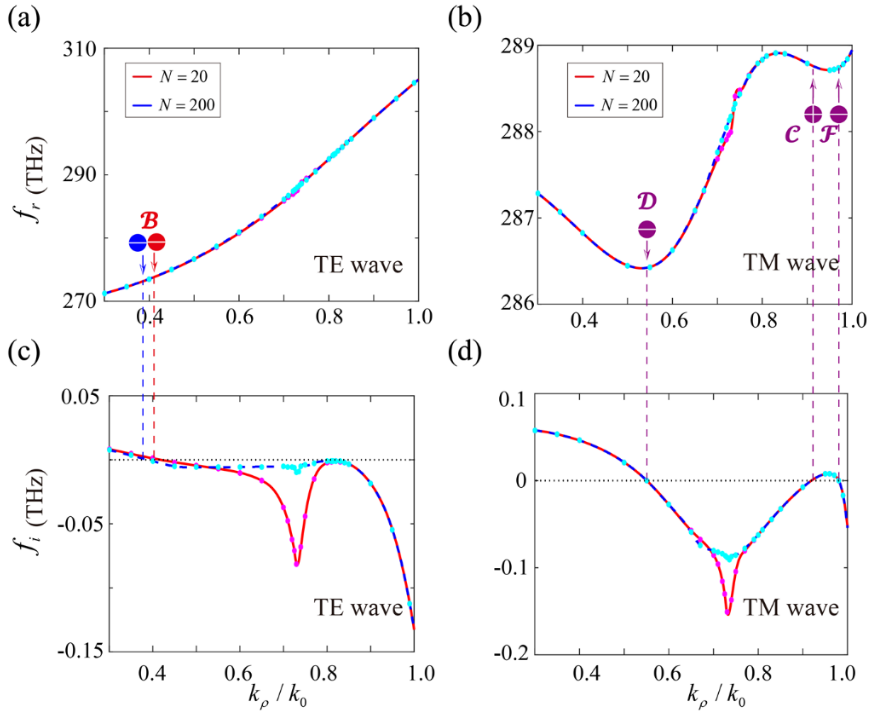

FIG. S15. Reflection-zero dispersion in the (a), (c) real frequency space, and (b), (d) imaginary frequency space for TE and TM waves. The positions of singularities with N=20 are indicated by red arrows, while those with N=200 are indicated by blue arrows. Here the layer D is in the lossy case.

In fact, systems with losses can be further connected to a wide region of non-Hermitian physics. More novel phenomena are waiting for discovery in the intersectional field of non-Hermitian and nodal semimetals [S19, S20]. In addition, a convenient method to realize similar singularity annihilations is through altering the thickness d_{E} of the silver layer E. With an increase of d_{E} from 15 nm to 20 nm to 25 nm, the reduced reflection phases at $k_\rho/k_0 = 0.8$ are highlighted on the cylinder by dark, moderate, and light red lines for both TE [Figs. S16(a)] and TM [Figs. S16(b)] waves. Such reductions lead to blueshifts of the edge states and movements of relevant singularities [Figs. S16(c)–S16(d)]. In this process, pairs of singularities originating from a common WNR, like $\mathcal{A}$ & $\mathcal{B}$ or $\mathcal{C}$ & $\mathcal{D}$, come away from each other. Tracing the evolution trajectory of +1 singularity $\mathcal{A}$ (red dotted lines), it approaches -1

singularity $\mathcal{E}$ (blue dotted lines), and vanishes after a collision with $d_{\mathrm{E}} = 21.6$ nm approximately. As is shown in Fig. S16(e), this annihilation happens near the point $f_0 = 293.31$ THz, $k_{\rho 0}/k_0 = 0.81$ (black cross), which verifies the conservation of topological charges in this system.

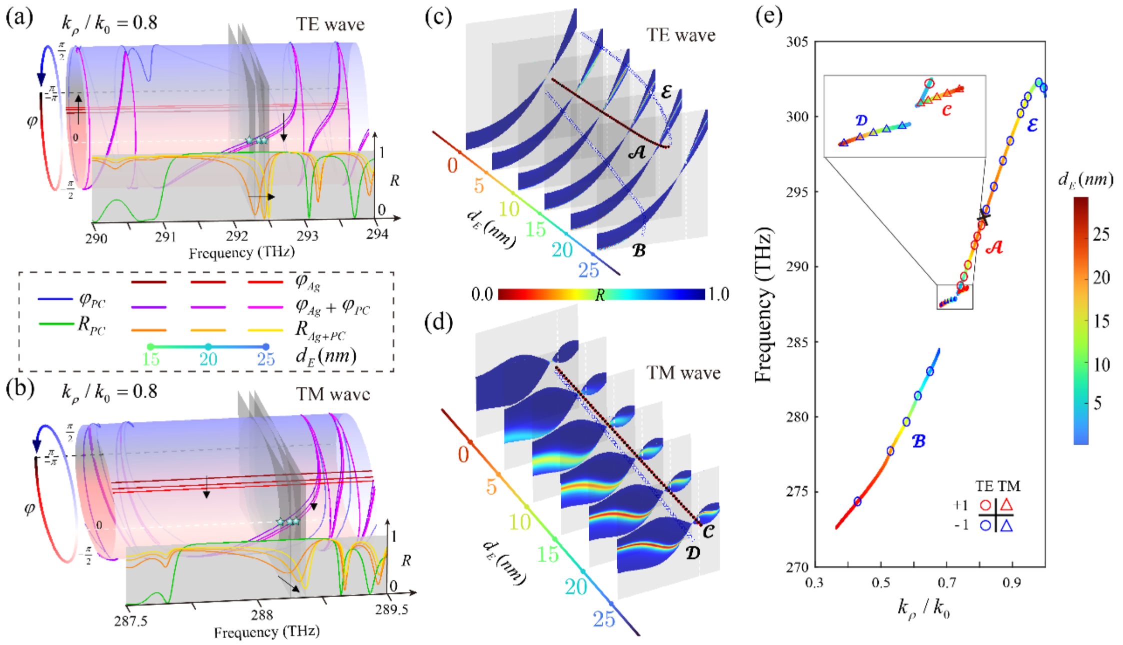

FIG. S16. Topological charge annihilation by altering the thickness of the silver layer d_{E}. When $k_\rho/k_0 = 0.8$ for the TE wave (a) and TM wave (b), the reflection phases φ_{Ag} for the silver layer E (red), φ_{PC} for the PC $[(\mathrm{CGD})_{22}\mathrm{B}]_{20}$ (blue) together with their sum $\varphi_{\mathrm{Ag}} + \varphi_{\mathrm{PC}}$ (magenta) are illustrated on the cylinders, and related reflections R_{PC} (green) together with $\mathrm{R}_{\mathrm{Ag+PC}}$ (orange) are illustrated in the insets. Their colors are dark/moderate/bright for the cases of different thickness of the silver layer $d_{\mathrm{E}} = 15/20/25$ nm, respectively. The boundary of $\varphi_{\mathrm{r}} = \pi(-\pi)$ and the center of $\varphi_{\mathrm{r}} = 0$ are highlighted by black and white dotted lines, respectively. Points that meet the condition of $\varphi_{\mathrm{Ag}} + \varphi_{\mathrm{PC}} = 0$ are marked by cyan stars. For the TE wave (c) and TM wave (d), vertical slice figures are the reflection spectra with different d_E. The trajectories for $\mathcal{A}$ and $\mathcal{C}$ with +1 topological charge, and $\mathcal{B}$, $\mathcal{D}$ and $\mathcal{E}$ with -1 topological charge are highlighted by the red and blue dotted lines, respectively. (e) Detailed evolution trajectories of these singularities for $d_{\mathrm{E}} \in [1,\ 30]$ nm. Several momentous points corresponding to $d_{\mathrm{E}} = 5/10/15/20/25$ nm are marked by circles (TE) or triangles (TM) with the color red (+1 singularities) and blue (-1 singularities) , respectively. The point of annihilation between $\mathcal{A}$ and $\mathcal{E}$ is marked by the black cross. The inset illustrates the situations for the TM wave with an enlarged view.

Sec. IX. Description of electromagnetic parameters

In the main text, graphene is selected for the sheet G, and its surface conductivity can be described by the sum of intra-band and inter-band components [S21]:

$$\sigma_G = \sigma_{\text{intra}} + \sigma_{\text{inter}} = i\frac{e^2 k_B T}{\pi\hbar^2(\omega + i\tau^{-1})}\frac{E_F}{k_B T} + 2i\frac{e^2 k_B T}{\pi\hbar^2(\omega + i\tau^{-1})} In\left(e^{-\frac{E_F}{k_B T}} + 1\right) + \mathrm{i}\frac{e^2}{4\pi\hbar} In\left(\frac{2|E_F| - \hbar(\omega + i\tau^{-1})}{2|E_F| + \hbar(\omega + i\tau^{-1})}\right) = \sigma_1 + \sigma_2 + \sigma_3, \quad \text{(S22)}$$

where $\tau = E_F/(e\upsilon_F^2)$ is the relaxation rate, and $E_F = \hbar\upsilon_f\sqrt{\pi|n|}$ is the Fermi energy. Here $\mu = 10^4\ \text{cm}^2/(\text{V}\cdot\text{S})$ and $\upsilon_f \approx 10^6$ m/s. Note that E_F can be modulated flexibly through electrostatic doping with tuning charge-carrier density n [S22]. Supposing that $\omega = 2\pi f = 2\pi \cdot 288$ THz and $T = 300$ K, the contributions of each part are plotted in Fig. S17. For $E_F > 0.4$ eV, it is reasonable to take the simplified mode $\sigma_G = \sigma_1 = i\frac{e^2 k_B T}{\pi\hbar(\omega + i\tau^{-1})}\frac{E_F}{k_B T}$. The refractive indexes of the layer B: BeO and the layer C: GaAs are marked by blue and orange lines, respectively.

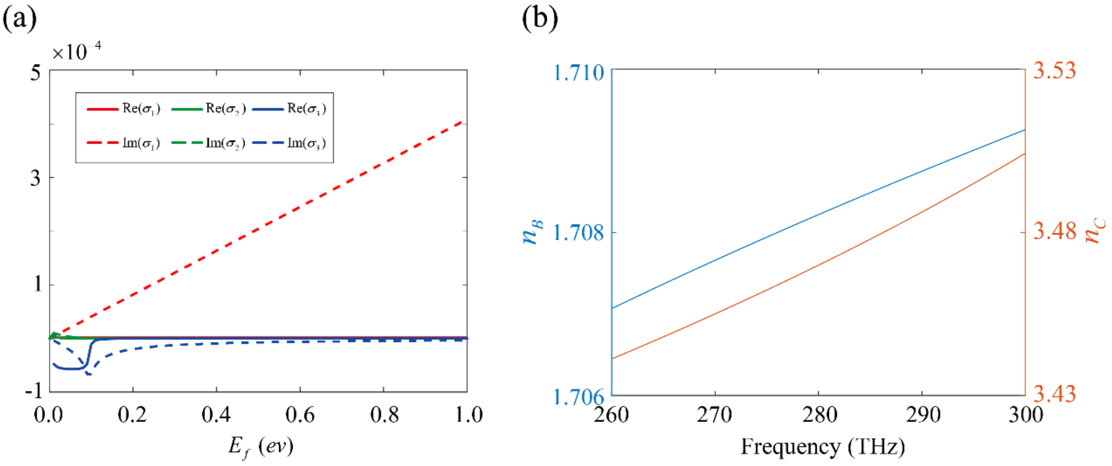

FIG. S17. (a) Contributions of each item in Eq. (S22), which describes the surface conductivity model of graphene. (b) The refractive indexes of the layer B: BeO and the layer C: GaAs.

Supplementary references

[S1] Tuz, V. R. Polaritons dispersion in a composite ferrite-semiconductor structure near gyrotropic-nihility state. *J. Magn. Magn. Mater.* **419**, 559–565 (2016).

[S2] Zhan, T. R., Shi, X., Dai, Y. Y., Liu, X. H. & Zi, J. Transfer matrix method for optics in graphene layers. *J. Phys.: Condens. Matter* **25**, 215301 (2013).

[S3] Vakil, A. & Engheta, N. Transformation optics using graphene. *Science* **332**, 1291–1294 (2010).

[S4] Hu, S. Y., Guo, Z. W., Jiang, H. T. & Chen, H. Photonic Dirac nodal-line

semimetals realized by a hypercrystal. *Phys. Rev. Res.* **4**, 023047 (2022).

[S5] Hu, M. Y., Zhang, Y., Jiang, X., Qiao, T., Wang, Q., Zhu, S. N., Xiao, M. & Liu, H. Double-bowl state in photonic Dirac nodal line semimetal. *Light Sci. Appl*. **10**, 170 (2021).

[S6] Deng, W. M., Chen, Z. M., Li, M. Y., Guo, C. H., Tian, Z. T., Sun, K. X., Chen, X. D., Chen, W. J. & Dong, J. W. Ideal nodal rings of one-dimensional photonic crystals in the visible region. *Light Sci. Appl.* **11**, 134 (2022).

[S7] Shi, X., Xue, C. H., Jiang, H. T. & Chen, H. Topological description for gaps of one-dimensional symmetric all-dielectric photonic crystals. *Opt. Express* **24**, 018580 (2016).

[S8] Lu, H. Z., Zhang, S.-B. & Shen, S.-Q. High-field magnetoconductivity of topological semimetals with short-range potential. *Phys. Rev. B* **92**, 045203 (2015).

[S9] Yang, B., Bi, Y. G., Zhang, R. X., Zhang, R. Y., You, O. B., Zhu, Z. H., Feng, J., Sun, H. B., Chan, C. T., Liu, C. X. & Zhang, S. Momentum space toroidal moment in a photonic metamaterial. *Nat. Commun.* **12**, 1784 (2021).

[S10] Sakotic, Z., Krasnok, A., Alú, A. & Jankovic, N. Topological scattering singularities and embedded eigenstates for polarization control and sensing applications. *Photon. Res*. **9**, 1310–1323 (2021).

[S11] Ermolaev, G., Voronin, K., Baranov, D. G., Kravets, V., Tselikov, G., Stebunov, Y., Yakubovsky, D., Novikov, S., Vyshnevyy, A., Mazitov, A., Kruglov, I., Zhukov, S., Romanov, R., Markeev, A. M., Arsenin, A., Novoselov, K. S., Grigorenko, A. N. & Volkov, V. Topological phase singularities in atomically thin high-refractive-index materials. *Nat. Commun*. **13**, 2049 (2022).

[S12] Kravets, V. G., Schedin, F., Jalil, R., Britnell, L., Gorbachev, R. V., Ansell, D., Thackray, B., Novoselov, K. S., Geim, A. K., Kabashin, A. V. & Grigorenko, A. N. Singular phase nano-optics in plasmonic metamaterials for label-free single-molecule detection. *Nat. Mater.* **12**, 304 (2013).

[S13] Sreekanth, K. V., Sreejith, S., Han, S., Mishra, A., Chen, X. X., Sun, H. D., Lim, C. T. & Singh, R. Biosensing with the singular phase of an ultrathin metal-dielectric nanophotonic cavity. *Nat. Commun.* **9**, 369 (2018).

[S14] Tsurimaki, Y., Tong, J. K., Boriskin, V. N., Semenov, A., Ayzatsky, M. I., Machekhin, Y. P., Chen, G. & Boriskinalb, S. V. Topological engineering of interfacial optical Tamm states for highly sensitive near-singular-phase optical detection. *ACS Photon*. **5**, 929–938 (2018).

[S15] Jia, X. Y., Hu, M., Soundarapandian, K., Yu, X. Q., Liu, Z. Y., Chen, Z. P., Narita, A., Mullen, K., Koppens, F. H. L., Jiang, J., Tielrooij, K. J., Bonn, M. & Wang, H. I. Kinetic ionic permeation and interfacial doping of supported graphene. *Nano Lett*. **19**, 9029–9036 (2019).

[S16] Bai, K., Fang, L., Liu, T.-R., Li, J.-Z., Wan, D. D. & Xiao, M. Nonlinearity-enabled higher-order exceptional singularities with ultra-enhanced signal-to-noise ratio. *Natl. Sci. Rev.* **10**, 259 (2023).

[S17] Monticone, F. & Alù, A. Embedded photonic eigenvalues in 3D nanostructures. *Phys. Rev. Lett.* **112**, 213903 (2014).

[S18] Novikov, V. B., Leontiev, A. P., Napolskii, K. S. & Murzina, T. V. Nonlocality-driven switchable fast-slow light effect in hyperbolic metamaterials in epsilon-near-zero regime. *Phys. Rev. B* **106**, 165415 (2022).

[S19] Zhen, B., Hsu, C. W., Igarashi, Y., Lu, L., Kaminer, I., Pick, A., Chua, S. L., Joannopoulos, J. D. & Soljacic, M. Spawning rings of exceptional points out of Dirac cones. *Nature* **525**, 354–358 (2015).

[S20] Liu, J. J., Li, Z. W., Chen, Z. G., Tang, W. Y., Chen, A., Liang, B., Ma, G. C. & Cheng, J. C. Experimental realization of Weyl exceptional rings in a synthetic three-dimensional non-Hermitian phononic crystal. *Phys. Rev. Lett.* **129**, 084301 (2022).

[S21] Hanson, G. W. Dyadic Green's functions and guided surface waves for a surface conductivity model of graphene. *J. Appl. Phys.* **103**, 064302 (2008).

[S22] Fan, Y. C., Wei, Z. Y., Li, H. Q., Chen, H. & Soukoulis, C. M. Photonic band gap of a graphene-embedded quarter-wave stack. *Phys. Rev. B* **88**, 241403 (2013).
